# ZMP recruits and excludes Pol IV–mediated DNA methylation in a site-specific manner

**DOI:** 10.1126/sciadv.adc9454

**Published:** 2022-11-25

**Authors:** Yuan Wang, Brandon H. Le, Jianqiang Wang, Chenjiang You, Yonghui Zhao, Mary Galli, Ye Xu, Andrea Gallavotti, Thomas Eulgem, Beixin Mo, Xuemei Chen

**Affiliations:** ^1^ Guangdong Provincial Key Laboratory for Plant Epigenetics, Longhua Institute of Innovative Biotechnology, College of Life Sciences and Oceanography, Shenzhen University, 518060 Shenzhen, China.; ^2^ Key Laboratory of Optoelectronic Devices and Systems of Ministry of Education and Guangdong Province, College of Optoelectronic Engineering, Shenzhen University, 518060 Shenzhen, China.; ^3^ Department of Botany and Plant Sciences, University of California, Riverside, CA 92521, USA.; ^4^ Institute for Integrative Genome Biology, University of California, Riverside, CA 92521, USA.; ^5^ State Key Laboratory of Genetic Engineering, School of Life Sciences, Institute of Plant Biology, Fudan University, Shanghai 200438, China.; ^6^ Academy for Advanced Interdisciplinary Studies, Nanjing Agricultural University, Nanjing 210095, China.; ^7^ Waksman Institute of Microbiology, Rutgers University, Piscataway, NJ 08854-8020, USA.

## Abstract

In plants, RNA-directed DNA methylation (RdDM) uses small interfering RNAs (siRNAs) to target transposable elements (TEs) but usually avoids genes. RNA polymerase IV (Pol IV) shapes the landscape of DNA methylation through its pivotal role in siRNA biogenesis. However, how Pol IV is recruited to specific loci, particularly how it avoids genes, is poorly understood. Here, we identified a Pol IV–interacting protein, ZMP (zinc finger, mouse double-minute/switching complex B, Plus-3 protein), which exerts a dual role in regulating siRNA biogenesis and DNA methylation at specific genomic regions. ZMP is required for siRNA biogenesis at some pericentromeric regions and prevents Pol IV from targeting a subset of TEs and genes at euchromatic loci. As a chromatin-associated protein, ZMP prefers regions with depleted histone H3 lysine 4 (H3K4) methylation abutted by regions with H3K4 methylation, probably monitoring changes in local H3K4 methylation status to regulate Pol IV’s chromatin occupancy. Our findings uncover a mechanism governing the specificity of RdDM.

## Teaser

ZMP enables RNA-directed DNA methylation at pericentromeric repeats and prevents it from euchromatic regions.

## INTRODUCTION

Transposable elements (TEs) are silenced via DNA methylation or histone H3 lysine 9 methylation (H3K9me) to maintain genome stability. In plants, RNA-directed DNA methylation (RdDM) uses small interfering RNAs (siRNAs) as guides to achieve sequence specificity. Similarly, piwi-interacting RNAs guide H3K9me or DNA methylation at TEs in insects and mammals (*1*). RdDM is responsible for de novo DNA methylation in all sequence contexts (CG, CHG, and CHH, where H = A, T, or C), while DNA methyltransferases such as methyltransferase 1 (MET1) and chromomethylase 3 (CMT3) maintain DNA methylation at CG and CHG contexts, respectively (*2*).While TEs exhibit DNA methylation in all three sequence contexts, genes are devoid of CHG and CHH methylation. A family of DNA demethylases removes DNA methylation from a subset of genes (*3*–*5*). The histone demethylase increase in bonsai methylation 1 (IBM1) removes H3K9me2 from the bodies of some genes to prevent CHG methylation by the DNA methyltransferase CMT3 (*6*–*8*). Although these mechanisms prevent DNA methylation at certain genes, they do not act on RdDM per se. In the current model, RdDM has a crucial role in determining the genomic DNA methylation landscape. De novo DNA methylation is initiated by polymerase II (Pol II)/RDR6 (RNA-dependent RNA polymerase 6)–mediated noncanonical RdDM (*9*). Once the initial heterochromatic marks are established, canonical Pol IV–dependent RdDM (Pol IV RdDM, hereafter referred to as RdDM) is probably recruited through these marks and reinforces DNA methylation. The activity of RdDM is particularly notable at smaller and younger TEs in euchromatic regions (*10*–*12*). However, how RdDM target loci are precisely specified, particularly how Pol IV RdDM is excluded from genes or prevented from spreading into genes from nearby TEs, is unknown.

RdDM begins with the transcription of target loci by RNA Pol IV.The transcripts are converted to double-stranded RNAs that are processed into 24–nucleotide (nt) siRNAs, which in turn direct the DNA methyltransferase domains rearranged methyltransferase 2 (DRM2) to homologous genomic loci for DNA methylation (*13*). Thus, the selection of Pol IV targets defines the profiles of 24-nt siRNAs and, consequently, the RdDM landscape in the genome (*14*, *15*). Two classes of genes, *SAWADEE HOMEODOMAIN HOMOLOGUE 1* (*SHH1*) and the *CLASSY* (*CLSY*) family, promote Pol IV’s chromatin occupancy at its genomic targets. SHH1 binds to H3K9me2 and unmethylated H3K4 (H3K4me0) through its tandem Tudor-like fold and is responsible for directing Pol IV to 44% of its genomic targets (*2*, *16*–*18*). The CLSY family of putative chromatin remodelers comprises four members that are, in aggregate, responsible for siRNA generation at nearly all Pol IV target loci (*19*), presumably via easing the passage of Pol IV through nucleosome remodeling (*13*). The four CLSY proteins aid Pol IV in a locus-specific manner: CLSY1 and CLSY2, similar to SHH1, act in euchromatic regions, whereas CLSY3 and CLSY4 are responsible for Pol IV–dependent siRNA production at pericentromeric heterochromatin independently of SHH1 (*19*).

Pol IV’s recruitment to targets, while crucial in determining the genomic landscape of RdDM and TE silencing, remains poorly understood. Pol IV generates abundant siRNAs from pericentromeric regions, but SHH1 is not required for the recruitment of Pol IV to these regions (*16*). In euchromatic regions, how Pol IV is prevented from targeting genes is unknown. Here, we report the roles of a Pol IV–interacting protein, ZMP [zinc finger, mouse double-minute/switching complex B (MDM/SWIB), Plus-3 protein], in regulating Pol IV–dependent siRNA biogenesis. ZMP is required for siRNA biogenesis at a subset of Pol IV targets that are located in pericentromeric and euchromatic regions and are independent of SHH1. ZMP also prevents Pol IV from targeting a set of genes in euchromatic regions, particularly genes that are lowly expressed and near TEs. As a chromatin-associated protein, ZMP achieves these effects through regulation of Pol IV’s chromatin occupancy. In vitro, the zinc finger [plant homeodomain (PHD)] of ZMP binds histone H3 tails with or without H3K4me but prefers H3K4me0. In vivo, ZMP’s chromatin-binding sites exhibit depleted H3K4me abutted by regions with H3K4me3. These chromatin features may underlie the different effects of ZMP toward Pol IV at different genomic locations. Genes protected by ZMP from Pol IV are enriched in those involved in pathogen responses, and ZMP’s suppression of Pol IV is essential for plant defense against an oomycete pathogen.

## RESULTS

### ZMP is a previously unknown Pol IV–interacting protein

**Fig. 1.**
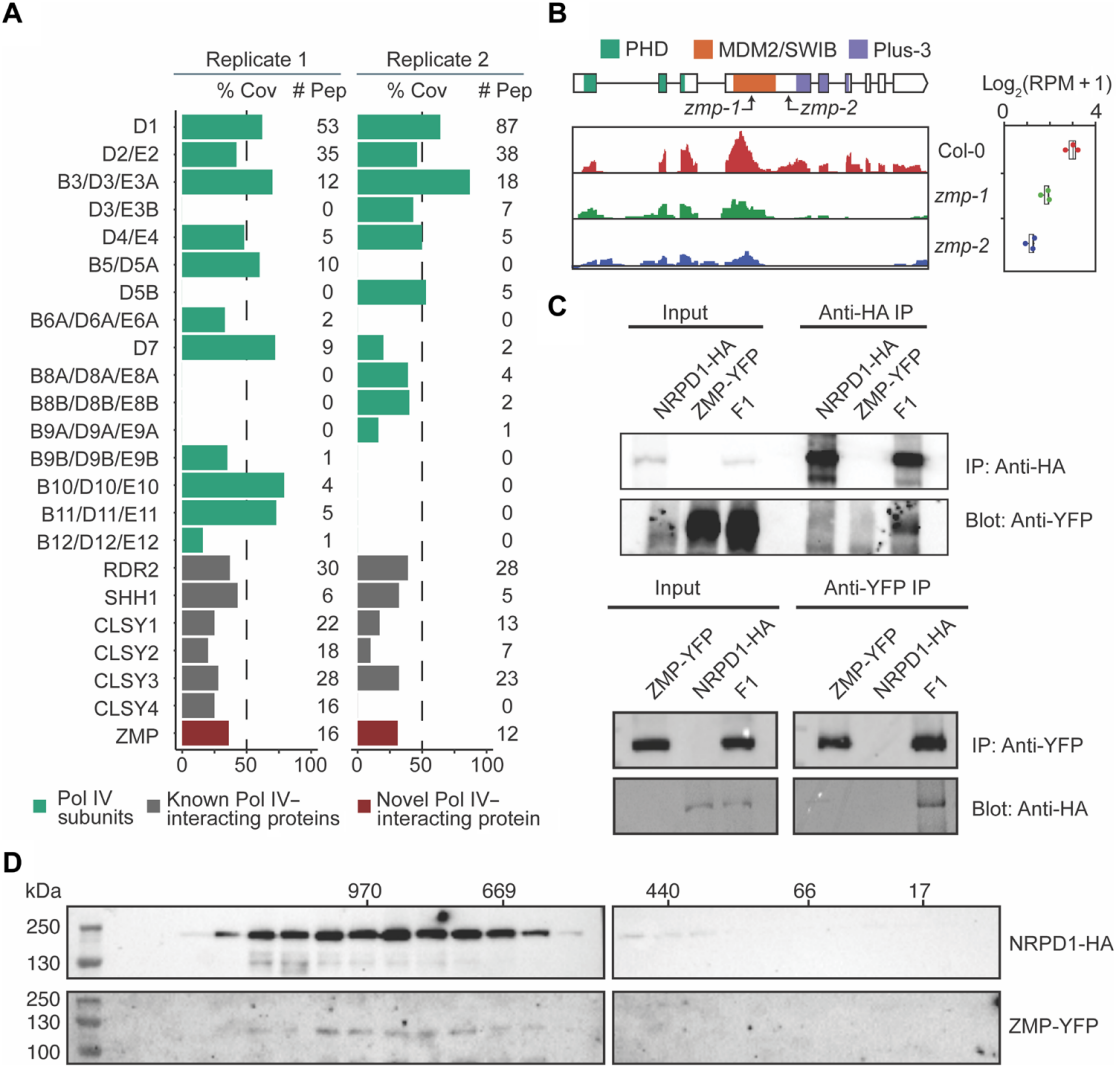
ZMP is an NRPD1-interacting protein. (**A**) Identification of ZMP from two independent replicates of tagged NRPD1 ChIP-MS in inflorescences. % Cov, percent coverage of the proteins by the identified peptides in MS; # Pep, number of distinct peptides identified for each indicated protein. (**B**) Schematic depiction of the *ZMP* gene overlayed with the predicted protein domains (top). *ZMP* transcripts in wild-type (WT) seedlings (Col-0) and two *zmp* alleles were determined by RNA sequencing (RNA-seq), visualized in Integrative Genomics Viewer (IGV) (bottom left), and quantified as boxplots (bottom right). The boxes and lines in the diagram represent exons and introns, respectively. Plus-3, a domain harboring highly conserved, three positively charged amino acid residues (arginine, arginine, and lysine). (**C**) Confirmation of NRPD1-HA and ZMP-YFP interaction by coimmunoprecipitation (co-IP) analysis. Lanes containing proteins extracted from the two parental lines (*NRPD1-HA* and *ZMP-YFP*) and the F1 lines from a cross of the two parental lines are indicated below the “input” and “immunoprecipitation (IP)” headings. The gel panels represent Western blots of input and IP samples. The IP in the top panel was done with anti-HA antibody to pull down NRPD1-HA, and the reciprocal IP in the bottom panel was performed with green fluorescent protein (GFP) antibody to pull down ZMP-YFP. The Western blots were done with anti-GFP and anti-HA antibodies separately. (**D**) Gel filtration chromatography assay showing that ZMP and NRPD1 codistribute in high–molecular weight fractions in vivo. Protein lysate from F1 lines expressing NRPD1-HA and ZMP-YFP was fractionated, eluted, and analyzed by Western blotting. The numbers on top mark the molecular weight of eluted fractions.

We expect proteins that recruit Pol IV to chromatin to be associated with Pol IV, particularly on chromatin. To identify Pol IV interaction partners on chromatin, we used FLAG and hemagglutinin (HA) epitope–tagged versions of the largest Pol IV subunit, nuclear RNA polymerase D1 (NRPD1)–FLAG and NRPD1-HA (*17*), to capture Pol IV transcription complexes by chromatin immunoprecipitation coupled with mass spectrometry (ChIP-MS) in two independent replicates (*20*, *21*). Unique peptides from NRPD-FLAG or NRPD-HA ChIP-MS were compared to those from the nontransgenic control [Columbia-0 (Col-0)]. In inflorescence tissue, nearly all Pol IV subunits (Fig. 1A and table S1) were identified by NRPD-FLAG or NRPD-HA ChIP-MS, similar to results from previous NRPD1-affinity purification (*17*, *22*, *23*). Known Pol IV–associated proteins including RDR2, SHH1, and the CLSY protein family were detected with relatively high peptide coverage, indicating the success of our assays (Fig. 1A and table S1). A previously unknwon, putative NRPD1-interacting protein, ZMP (AT5G63700) (Fig. 1, A and B, and table S1), was identified in both replicates.

To confirm the ZMP–Pol IV interaction, we carried out coimmunoprecipitation (co-IP) using F1 transgenic plants containing NRPD1-HA and ZMP–yellow fluorescent protein (YFP) expressed from their native promoters. NRPD1-HA was immunoprecipitated with anti-HA antibodies, and both NRPD1-HA and ZMP-YFP proteins were detected in the immunoprecipitate (Fig. 1C, top). A reciprocal co-IP experiment was also performed by pulling down ZMP-YFP, and NRPD1-HA was coimmunoprecipitated (Fig. 1C, bottom), thus confirming the in vivo interactions between ZMP and NRPD1. In a complementary experiment, extracts from F1 transgenic plants expressing both ZMP-YFP and NRPD1-HA were subjected to size exclusion chromatography (Fig. 1D). ZMP-YFP cofractionated with NRPD1-HA almost completely in high molecular weight fractions, consistent with their in planta association (Fig. 1D).

There are five ZMP-like proteins in *Arabidopsis* that contain at least two of the three domains. A phylogenetic analysis of ZMP-like proteins from land plants suggested that the last common ancestor of ZMP-like proteins in multicellular plants contains four protein domains: zinc finger, MDM2/SWIB, Plus-3, and glycine-tyrosine-phenylalanine (GYF) (fig. S1A). During evolution, some ancestral proteins lost certain domains. For instance, the ancestor of ZMP in the latest common ancestor of rosids lost the GYF domain (fig. S1A). In *Arabidopsis*, ZMP-like proteins clustered together and separately from most MDM2/SWIB domain–containing proteins, probably due to the presence of domains other than MDM2/SWIB (fig. S1B). The functions of ZMP-like proteins may also have diverged with the protein domain reorganization, an example being NERD (needed for RDR2-independent DNA methylation) with a connection to argonaute 2 (AGO2) and 21-nt siRNAs (*24*).

### *ZMP* promotes the biogenesis of Pol IV–dependent, 24-nt siRNAs at a subset of loci

To determine whether ZMP, as a Pol IV–interacting protein, plays a role in siRNA biogenesis, we first obtained two T-DNA insertion alleles (*zmp-1* and *zmp-2*) in *ZMP*. These mutants showed no obvious developmental phenotypes. RNA sequencing (RNA-seq) with the mutants revealed reduced levels of *ZMP* transcripts, with little or no reads detected 3′ to the T-DNA insertion sites (Fig. 1B). The *zmp-2* allele showed lower levels of *ZMP* transcripts and was selected for further functional analysis (Fig. 1B).

Next, we carried out small RNA-seq (sRNA-seq) with wild-type (WT), *zmp-2*, *nrpd1-3*, and *zmp-2 ZMP-YFP*, a *zmp-2* mutant harboring a *ZMP-YFP* transgene driven by the *ZMP* promoter. Three biological replicates generated ~15 to 30 million reads per library and yielded a high degree of reproducibility (fig. S2 and dataset S1). The global size distribution of sRNAs showed a marked reduction in 24-nt sRNAs in the *nrpd1-3* mutant, as expected. The *zmp-2* mutant did not show an obvious reduction in 24-nt sRNAs (fig. S3A), suggesting that *ZMP* does not have a globally strong impact on siRNA biogenesis.

**Fig. 2.**
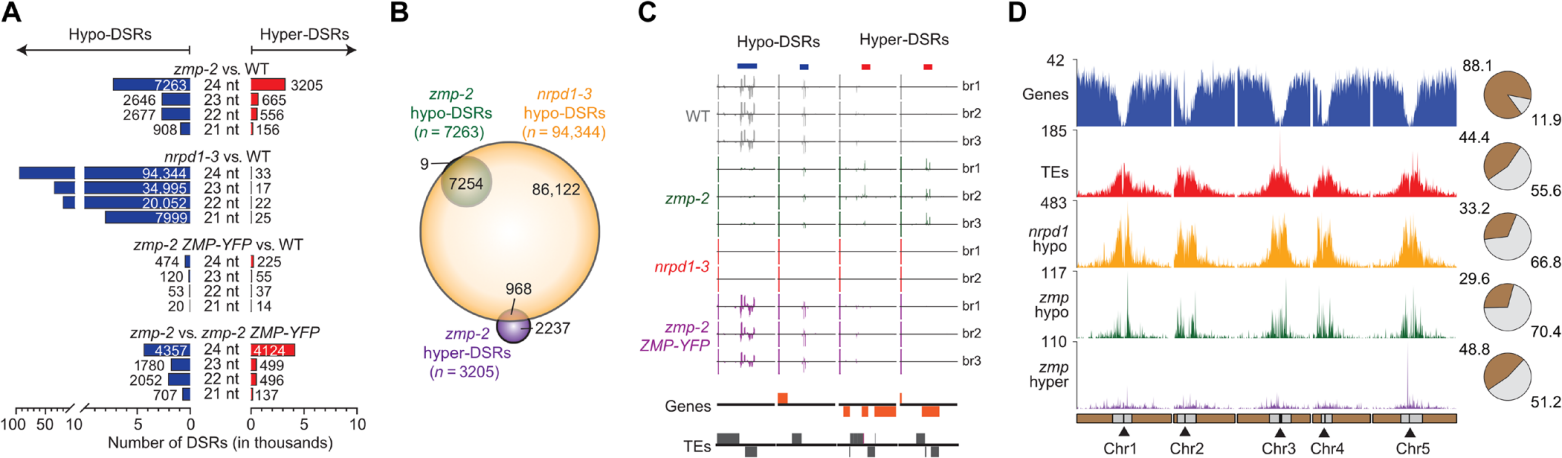
ZMP promotes and represses the biogenesis of siRNAs at different genomic loci. (**A**) DSRs for each size class between genotypes as indicated. The genome was tiled into 100–base pair (bp) nonoverlapping windows, and ribosomal RNA (rRNA)–normalized sRNA abundance was compared between genotypes for each window. Hypo- and hyper-DSRs denote regions with reduced or increased sRNAs [edgeR, fold change (FC) ≥ 2, false discovery rate (FDR) ≤ 0.05; see Materials and Methods]. (**B**) Overlap analysis of 24-nt *nrpd1* hypo-DSR, *zmp* hypo-DSR, and *zmp* hyper-DSR. (**C**) Genome browser view showing 24-nt siRNA abundance at representative 24-nt *zmp* hypo- and hyper-DSR loci in WT, *zmp-2*, *nrpd1-3*, and *zmp-2 ZMP-YFP*. Each track represents a biological replicate (br) where the signals above or below the black line indicate sRNAs from the sense and antisense strands, respectively. Genes and TEs along these regions are depicted by orange and gray boxes, respectively, with orientation indicated by boxes above (sense) or below (antisense) the black line. The blue and red bars indicate the hypo- and hyper-DSRs, respectively. Location identifiers and signal scales (in parentheses) for these regions include, from left to right, AT1TE67625 (−600 to 600), AT2TE52920 (−1200 to 1200), AT2G20465 (−30 to 30), and AT4G11485 (−50 to 50). (**D**) DSR distribution along the genome. Numbers of genome features (genes and TEs) and 24-nt DSR loci (*zmp* hypo, *zmp* hyper, and *nrpd1* hypo) in 100-kb nonoverlapping windows along the chromosomes were plotted. The pie charts show proportions of the genome features and 24-nt DSRs located on euchromatic (brown) and pericentromeric (gray) regions. Centromeres and pericentromeric regions for each chromosome are indicated by the triangles and gray rectangles, respectively.

**Fig. 3.**
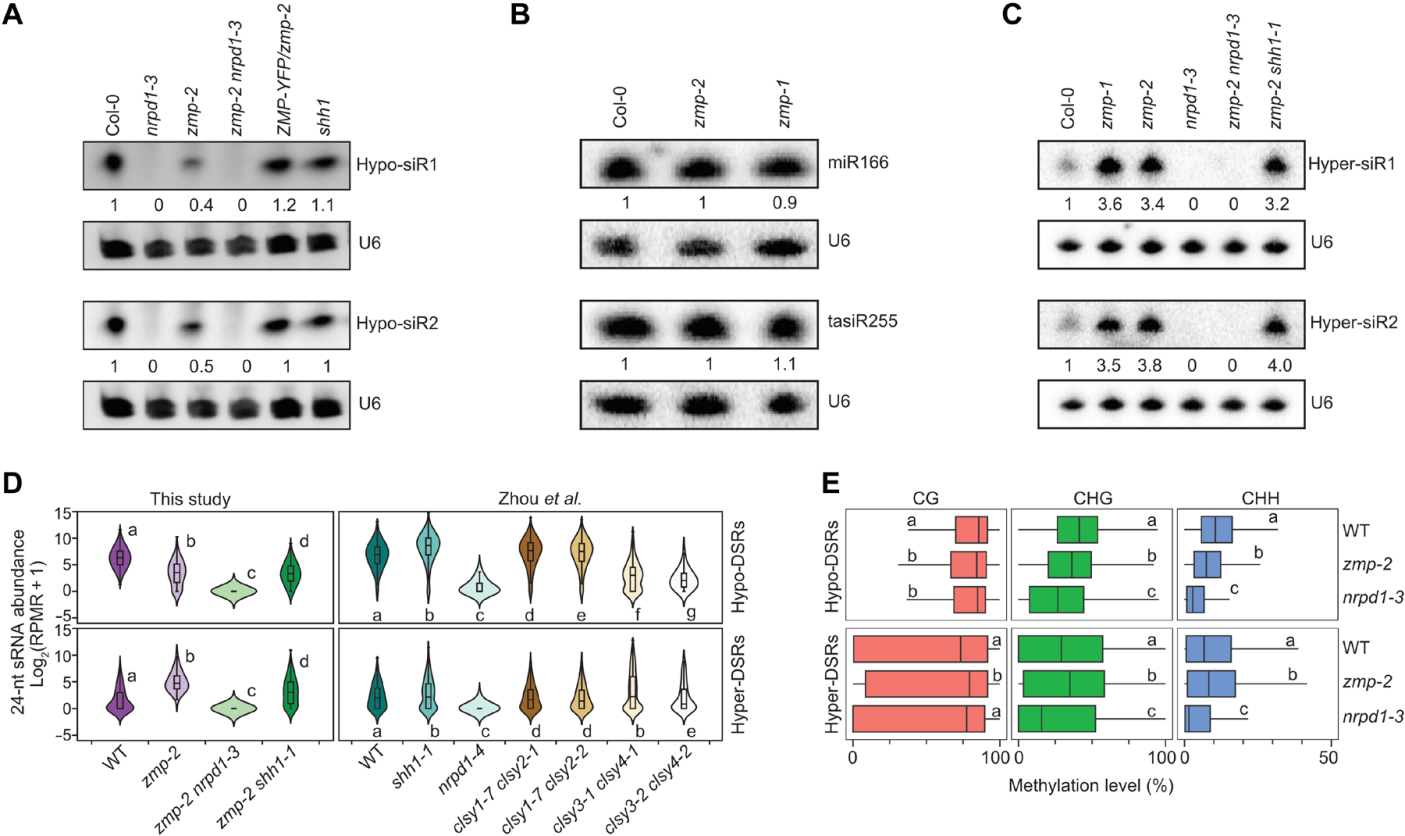
*ZMP* promotes Pol IV–dependent siRNAs from *SHH1*-indepednent regions. (**A** to **C**) RNA gel blot analysis of two 24-nt *zmp* hypo-DSRs (A), an miRNA (miR166), a ta-siRNA [tasiR255 (TAS1)] (B), and two 24-nt *zmp* hyper-DSRs (C) using RNA extracted from the indicated genotypes with U6 as an internal control. Numbers represent relative abundance. (**D**) Violin and box plots showing 24-nt siRNA abundance at *zmp* hypo-DSR (top) and hyper-DSRs (bottom) in the genotypes indicated below. The data from Zhou *et al.* (*19*) and this study were plotted in the right and left panels, respectively. Statistical significance was determined by the Wilcoxon rank sum test with the Holm correction for multiple comparisons and represented by letters (a to g; where the same letter indicates no significant differences between genotypes in pairwise comparisons). RPMR, reads per million rRNA fragments. (**E**) DNA methylation level distribution at 24-nt *zmp* hypo- and hyper-DSRs in WT, *zmp-2*, and *nrpd1-3*, in all sequence contexts where H = A, C, or T. Statistical significance [represented by the letters (a to c)] was determined by the Student’s *t* test and the Bonferonni-Hochberg correction for multiple testing. Genotypes with the same letters show no significant differences in pairwise comparisons.

To determine whether *ZMP* affects siRNA biogenesis at specific loci, we searched for differential sRNA regions (DSRs) between WT and each of the other three genotypes (*zmp-2*, *nrpd1-3*, and *zmp-2 ZMP-YFP*) (see Materials and Methods). As expected, 94,344 hypo-DSRs (i.e., regions with statistically significant reduction in siRNA accumulation) for 24-nt siRNAs were found in the *nrpd1* mutant, representing regions that produce Pol IV–dependent 24-nt siRNAs (Fig. 2A and dataset S2). In the *zmp-2* mutant, 7263 24-nt hypo-DSRs were identified, and these *zmp* hypo-DSRs nearly completely overlapped with *nrpd1* hypo-DSRs (Fig. 2, A to C, and dataset S2). The genomic distribution of *zmp* hypo-DSRs and *nrpd1* hypo-DSRs was similar, showing enrichment at pericentromeric regions (Fig. 2D). DSR analysis between WT and *zmp-2 ZMP-YFP* or between *zmp-2* and *zmp-2 ZMP-YFP* showed that *ZMP-YFP* largely rescued the defects of *zmp-2* in siRNA biogenesis (Fig. 2, A and C). Northern blotting further confirmed a reduction of Pol IV–dependent 24-nt siRNAs at two genomic loci in *zmp-2*, which was then recovered by introducing the functional *ZMP* gene (Fig. 3A). Moreover, *ZMP* does not influence the expression of known Pol IV interactors and RdDM components, which argues against the possibility of indirect effects of *ZMP* on siRNA production (fig. S3C). Collectively, these results show that *ZMP* promotes the biogenesis of Pol IV–dependent siRNAs at a subset of Pol IV–dependent loci. The levels of microRNAs (miRNAs) and trans-acting siRNAs (ta-siRNAs) were unchanged in *zmp* mutants (Fig. 3B and fig. S3B). Therefore, *ZMP* acts exclusively with Pol IV for siRNA production, rather than assisting Pol II in the production of miRNAs and ta-siRNAs.

At present, only two classes of genes are known to promote the production of Pol IV–dependent siRNAs in a locus-specific manner as does *ZMP*, and these are *SHH1* and the *CLSY* family. To determine whether *ZMP* and *SHH1* act at the same or different loci to promote Pol IV–dependent siRNA production, we first used public sRNA datasets (*19*) to examine siRNA levels at the *ZMP*-dependent loci (i.e., *zmp* hypo-DSRs) in *nrpd1* and *shh1* mutants. The levels of 24-nt siRNAs at *zmp* hypo-DSRs were nearly absent in *nrpd1-4* but were slightly increased in *shh1* (Fig. 3D, top right), suggesting that *SHH1* does not contribute to siRNA biogenesis at regions that require *ZMP*. To confirm this, we generated a *zmp-2 shh1-1* double mutant and performed sRNA-seq with this double mutant as well as WT, *zmp-2*, and *zmp-2 nrpd1-3*. At the *zmp* hypo-DSRs, as expected, the levels of 24-nt siRNAs were reduced in *zmp-2* as compared to WT and were nearly absent in *zmp-2 nrpd1-3*. However, 24-nt siRNA levels were comparable between *zmp-2* and *zmp-2 shh1* (Fig. 3D, top left) at these loci, confirming that *SHH1* is largely dispensable for siRNA biogenesis at regions dependent on *ZMP*. We also validated this by Northern blot analysis at two *zmp* hypo-DSR loci (Fig. 3A). Using sRNA datasets of *clsy12* and *clsy34* double mutants (*19*), we found that the levels of 24-nt siRNAs at *zmp* hypo-DSRs were reduced in the *clsy34* double mutant but unaffected in the *clsy12* double mutant (Fig. 3D, top right). This is consistent with the enrichment of *zmp* hypo-DSRs in pericentromeric regions, where *CLSY3*/*4* are known to act (Fig. 3D) (*19*). Together, these findings demonstrate that *ZMP* promotes Pol IV–dependent siRNA biogenesis at a subset of genomic regions where *CLSY3*/*4*, but not *CLSY1*/*2* and *SHH1*, is required.

To evaluate the effects of *ZMP* on DNA methylation, we performed methylome profiling with WT, *zmp-2*, and *nrpd1-3*, each with two biological replicates, which were highly reproducible (fig. S4). The *nrpd1* mutant showed a large reduction in DNA methylation at TEs, particularly at CHG and CHH sequence contexts, but was unaffected in gene body methylation, which is consistent with the known role of RdDM (fig. S5, A and B) (*15*). Only a small reduction in CHG and CHH methylation was found globally at TEs in the *zmp-2* mutant (fig. S5A). At *zmp* hypo-DSRs, in general, and at TEs overlapped with *zmp* hypo-DSRs, specifically, the levels of DNA methylation were significantly reduced (Fig. 3E and fig. S5, C and D), consistent with reduced levels of 24-nt siRNAs. By contrast, randomly selected TEs that do not overlap with the *zmp* hypo-DSRs show no changes in DNA methylation (fig. S5, C and D). Collectively, *ZMP* promotes the biogenesis of Pol IV–dependent siRNAs and DNA methylation at a fraction of Pol IV target sites, and *ZMP*-dependent regions are enriched at pericentromeric heterochromatin.

### *ZMP* recruits Pol IV to a subset of Pol IV–dependent siRNA loci

**Fig. 4.**
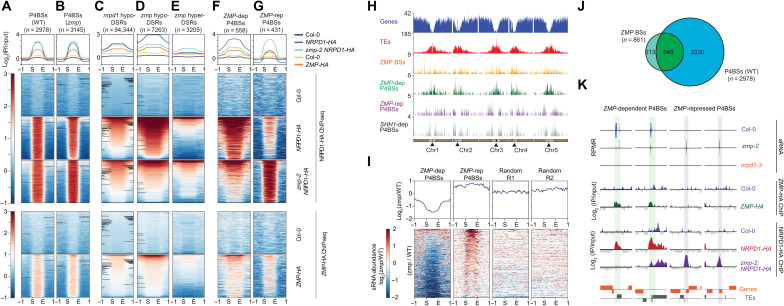
*ZMP* facilitates and prevents Pol IV’s chromatin association at *zmp* hypo- and hyper-DSRs, respectively. (**A** and **B**) NRPD1 and ZMP ChIP signals at all P4BSs in WT (A) and *zmp* (B) backgrounds. (**C** to **E**) NRPD1 and ZMP ChIP signals at *nrpd1* hypo-DSRs (C), *zmp* hypo-DSRs (D), and *zmp* hyper-DSRs (E). (**F** and **G**) NRPD1 and ZMP ChIP signals at defined P4BSs: *ZMP*-dep P4BSs (F) and *ZMP*-rep P4BSs (G). (**H**) Genome-wide distribution of ZMP and P4BSs (as indicated in the left) along the chromosome in 100-kb windows. Centromeres, euchromatic, and pericentromeric regions are indicated by the triangles, brown rectangles, and gray rectangles, respectively. Numbers along the *y* axis indicate the numbers of sites per 100-kb windows. (**I**) Relative abundance of 24-nt siRNAs at *ZMP*-dep and *ZMP*-rep P4BSs. Random sites were generated with similar size and numbers as the *ZMP*-dep and *ZMP*-rep P4BSs. All biological replicates of WT and *zmp-2* sRNA-seq data were merged and plotted. Heatmaps and profile plots show normalized signals in (A) to (G) (log_2_ ratios of IP/input) and in (I) (log_2_ ratios of *zmp-2*/WT) at the start (S) and end (E) of the indicated regions and 1-kb upstream (−1) and downstream (1). (**J**) Overlap analysis of P4BSs and ZMP-binding sites (ZMP BSs) identified from NRPD1-HA and ZMP-HA ChIP-seq datasets. (**K**) Genome browser views showing 24-nt sRNAs, ZMP-HA, and NRPD1-HA signal abundance at selected P4BSs defined in (F) and (G). Vertical colored bars spanning all tracks highlight the P4BSs. Genes and TEs along these regions are depicted by orange and gray boxes, respectively, with orientation indicated by boxes above (sense) or below (antisense) the line. Scales for the sRNA-seq tracks are from −100 to 100. Scales for the ChIP-seq tracks are from −1 to 4. Features associated with these regions are, from left to right, AT3TE15400, AT1TE55560, AT3G07775, and AT5G43755.

As ZMP was found as a Pol IV–interacting protein on chromatin, it is possible that the role of *ZMP* in the biogenesis of 24-nt siRNAs at *zmp* hypo-DSRs lies in the chromatin recruitment or maintenance of Pol IV. To test this hypothesis, we determined genome-wide Pol IV occupancy in *NRPD1-HA* and *zmp-2 NRPD1-HA* backgrounds (fig. S6A) via ChIP sequencing (ChIP-seq) using an anti-HA antibody; Col-0 plants without NRPD1-HA were used as a negative control. Two biological replicates were reproducible (fig. S7A), and NRPD1-HA peaks common in the two biological replicates were defined as high-confidence Pol IV–binding sites (P4BSs). P4BSs (2978 and 3145) with a high degree of overlap were found in WT and *zmp-2*, respectively (Fig. 4, A and B, fig. S6B, and dataset S3). Moreover, Pol IV ChIP signals were enriched at *nrpd1* hypo-DSRs in both WT and *zmp* backgrounds (Fig. 4C), suggesting that the Pol IV ChIP-seq was successful. *zmp* hypo-DSRs largely overlapped with P4BSs in WT (fig. S6C) and *nrpd1* hypo-DSRs (Fig. 2B), consistent with the role of both *ZMP* and Pol IV in siRNA biogenesis at these sites. However, NRPD1-HA ChIP signals were not obviously different between WT and *zmp-2* at the 2978 P4BSs (Fig. 4A and dataset S3), suggesting that *ZMP* is not required for Pol IV occupancy at most genomic loci. Thus, we further determined *ZMP*’s contribution to Pol IV’s chromatin occupancy at specific sites, especially at *zmp* hypo-DSRs. NRPD1-HA ChIP signals were greatly reduced at *zmp* hypo-DSRs in *zmp-2* (Fig. 4D), suggesting that *ZMP* contributes to siRNA generation by affecting Pol IV’s chromatin occupancy. To further confirm this, we examined siRNA levels at regions differentially occupied by Pol IV between WT and *zmp*. Using the biological replicates, we determined differentially enriched NRPD1-HA peaks between WT and *zmp-2* (see Materials and Methods) and identified 558 P4BSs enriched in WT versus *zmp-2*, which were defined as *ZMP*-dependent P4BSs (*ZMP*-dep P4BSs; Fig. 4F and dataset S3). These *ZMP*-dep P4BSs showed enrichment at pericentromeric regions similar to that of TEs (Fig. 4H) and *zmp* hypo-DSRs (Fig. 2D). siRNA levels were reduced in *zmp-2* compared to WT at *ZMP*-dep P4BSs (Fig. 4, I and K, and fig. S8). These results together suggested that *ZMP* is required for Pol IV’s occupancy at specific sites for the biogenesis of 24-nt siRNAs. *ZMP*-dep P4BSs and *SHH1*-dep P4BSs (*16*) showed little overlap (fig. S6D), further confirming that *ZMP* functions independently of *SHH1*.

We next asked whether ZMP binds to chromatin to mediate Pol IV’s chromatin occupancy at the *ZMP*-dep P4BSs. We profiled genome-wide ZMP chromatin occupancy via ChIP-seq using a *ZMP-HA* transgenic line (WT being the negative control) in two biological repeats (fig. S7B). Reproducible ZMP-binding peaks (861) were identified, ~75% of which overlapped with P4BSs (Fig. 4J and dataset S3), but few overlapped with *SHH1*-dep P4BSs (fig. S6E). In particular, ZMP occupancy was found at *ZMP*-dep P4BSs (Fig. 4F). Collectively, ZMP directly binds to chromatin to allow for Pol IV occupancy at a subset of Pol IV target sites for siRNA biogenesis.

### ZMP prefers to bind H3K4me-depleted regions with adjacent H3K4me

To gain further insight into the mechanism by which ZMP recruits Pol IV to a subset of genomic regions, we investigated ZMP’s biochemical properties. The ZMP protein has three putative chromatin-binding motifs (Fig. 1B), including a classical 4× cysteine–histidine–3× cysteine (C4HC3)–type zinc finger PHD at the N terminus, a chromatin remodeler MDM2/SWIB domain in the middle and a Plus-3 domain at the C terminus. PHD, a 50– to 80–amino acid domain of diverse sequences and present in many chromatin-associated proteins; binds the N-terminal tail of histone H3 with specific methylation states at lysine 4 (such as H3K4me0 or H3K4me3) and translates this chromatin status into regulatory outputs (*25*). The MDM2/SWIB domain is a conserved region present in the human oncoprotein MDM2 that negatively regulates p53 expression and in BAF60b from the switch/sucrose non-fermentable (SWI/SNF) complex B that acts in chromatin remodeling and gene activation (*26*). The Plus-3 domain, named because of three positively charged amino acids, also resides in human RTF1 (Restore TBP function 1) and binds single-stranded DNA to play a role in the structural organization of the elongating transcription bubble, rather than in specific DNA sequence recognition (*27*).

As ZMP associates with chromatin, we first tested whether ZMP may recognize specific DNA motifs by searching for enriched sequence motifs at ZMP-HA ChIP peaks. No consensus DNA motif was found, suggesting that ZMP is not a sequence-specific DNA binding protein. Since ZMP has a PHD domain that might be responsible for the recognition of chromatin features, we then focused on studying whether the PHD domain determines ZMP’s chromatin distribution pattern.

**Fig. 5.**
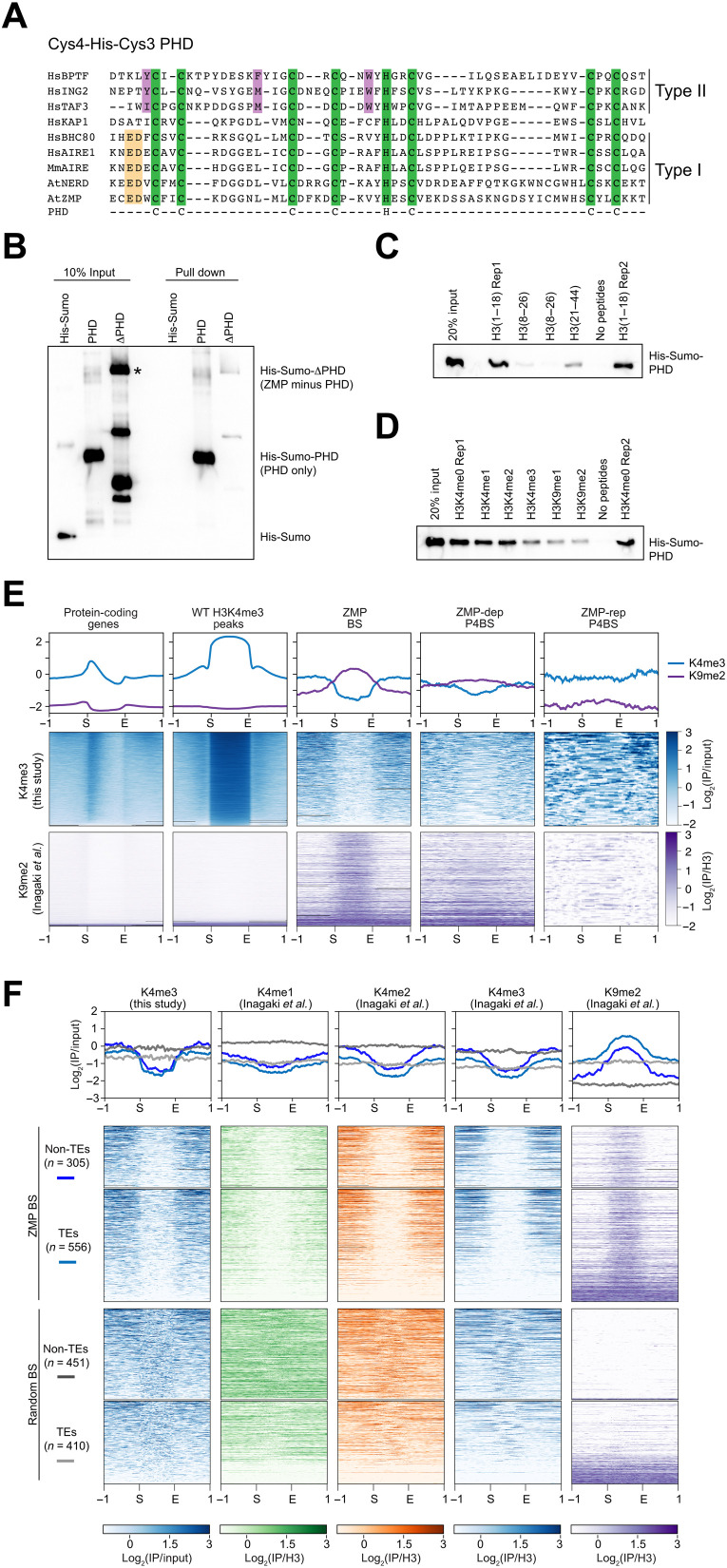
ZMP prefers H3K4me-depleted regions with adjacent H3K4 methylation. (**A**) Sequence comparison of ZMP PHD with other PHDs known to bind H3K4me0 (type I) and H3K4me3 (type II). HsKAP1 has a PHD domain but does not bind histone H3 (*79*). The green boxes highlight the conserved C4HC3 residues chelating zinc ions. Conserved residues in the type I and type II PHD fingers are highlighted in yellow and purple, respectively. At, *A. thaliana*; Mm, *Mus musculus*; Hs, *Homo sapiens*. GenBank accessions of the PHD-containing proteins are as follows: AtNERD, NP_179241.4; AtZMP, NP_201175.2; HsAIRE1, CAA0859.1; HsBHC80, NP_001095272.1; HsBPTF, Q12830.3; HsING2, CAC20567.1; HsKAP1, AAB37341.1; HsTAF3, XP_007933636.1; and MmAIRE, NP_033776.1. (**B**) In vitro binding assay using recombinant PHD of ZMP (PHD) and the ZMP protein without PHD (ZMP-ΔPHD) as preys and biotinylated histone H3 N-terminal tail peptides as the bait. His-SUMO served as a negative control. (**C** and **D**) In vitro binding assays of PHD of ZMP (PHD) with unmethylated histone H3 tail peptides (C) and H3K4me and H3K9me (D). Rep1 and Rep2, two repeats. (**E**) H3K4me3 and H3K9me2 signals at protein-coding genes, H3K4me3 peaks, ZMP-binding sites, and P4BSs (*ZMP*-dep and *ZMP*-rep). Heatmaps and profile plots show normalized signals (log_2_ ratios of IP/input or IP/H3) at the start (S) and end (E) of the indicated regions and 1-kb upstream (−1) and downstream (1). (**F**) Histone H3 profiles at ZMP-binding sites (ZMP-BSs) divided by sites overlapping or not overlapping with TE features. Random sites were generated with similar size and numbers as ZMP-BSs. Histone H3 datasets (except for H3K4me3 from this study) were downloaded from public repository (indicated on top), and IP signals were normalized relative to the H3 or input control.

Two distinct types of PHD have been experimentally defined to specifically bind either methylated H3K4 or H3K4me0 (Fig. 5A) (*28*–*30*). Type 2 recognizes H3K4me3, regarded as an epigenetic mark for transcription activation, through an aromatic cage (*29*). Type 1, devoid of the cage, uses an N-terminal aspartic acid (indicated in Fig. 5A) to recognize H3K4me0, perceived as a mark for transcription repression. Sequence alignment indicates that the ZMP PHD belongs to type 1, similar to PHDs of human Autoimmune Regulator (AIRE)-1, mouse AIRE, human BHC80 (BRAF–HDAC complex 80), and *Arabidopsis* NERD, in which the conserved acidic patch (E600 and D601) was previously implicated in H3K4me0 recognition (Fig. 5A) (*24*, *29*).

To confirm the predicted specificity of the PHD of ZMP (hereafter referred to as PHD), we conducted histone-binding assays using recombinant PHD and biotinylated histone H3 peptides (Fig. 5, B and C). The H3 tail peptide (1 to 18 amino acids) showed prominent binding to PHD but not the ZMP protein without the PHD region (ΔPHD) (Fig. 5, B and C). Similar assays with methylated H3 tails found that PHD was also able to bind H3K4me1, H3K4me2, and H3K4me3 but at reduced levels compared to H3K4me0 (Fig. 5D), indicating that ZMP prefers H3K4me0. As compared to the unmodified H3 peptide (1 to 18 amino acids; H3K4me0 and H3K9me0), H3K9me1 and H3K9me2 peptides were bound by PHD at reduced levels (Fig. 5D), suggesting that ZMP does not prefer these repressive marks, which are recognized by SHH1 (*2*, *16*, *18*).

We further sought for clues to ZMP’s binding preference in the genome. Since ZMP binds H3 tails with H3K4me0 better than those with H3K4me in vitro, we performed H3K4me3 ChIP-seq to determine the status of H3K4 methylation at ZMP-binding sites in vivo. Consistent with previous reports (*30*–*32*), H3K4me3 was found to be enriched near the transcription start sites of genes but depleted from TE regions (Fig. 5E and dataset S3). H3K4me3 signals were absent in ZMP-binding sites but present in adjacent regions both upstream and downstream, a feature that was also weakly displayed by *ZMP*-dep P4BSs (Fig. 5E). H3K9me2 showed the opposite profile in ZMP-binding sites and *ZMP*-dep P4BSs (Fig. 5E). These features were found for both TE and non-TE ZMP-binding sites (Fig. 5F). To examine the chromatin features of ZMP-binding sites further, we took advantage of existing epigenomic datasets (*33*, *34*). For this analysis, 861 randomly sampled genomic regions were included for comparison with the 861 ZMP-binding sites. Relative to the random sites, ZMP-binding sites (both in TE and non-TE) were depleted of the active marks H3K4me1, H3K4me2, and H3K4me3 and enriched in the repressive mark H3K9me2 (Fig. 5F). Furthermore, the adjacent regions of ZMP-binding sites enriched more H3K4me1, H3K4me2, and H3K4me3 than the ZMP-binding sites. This H3K4me distribution pattern over the ZMP-binding sites and adjacent regions was not observed in randomly selected sites (Fig. 5F). In summary, H3K4me-depleted regions with adjacent H3K4me3 are occupied by ZMP in vivo. This may reflect ZMP’s preference for H3K4me0 and tolerance of H3K4me3 in vitro (Fig. 5D).

### *ZMP* prevents Pol IV–dependent siRNA biogenesis from certain genes

A long-standing mystery is how Pol IV distinguishes TEs/repeats from genes. So far, no factor that prevents Pol IV from producing ectopic siRNAs is known. In the sRNA-seq experiments, we found that the *zmp-2* mutant produced more 24-nt siRNAs than WT at thousands of genomic sites (i.e., *zmp* hyper-DSRs) (Fig. 2A and dataset S2). The presence of hyper-DSRs in *zmp-2* was not due to a normalization issue caused by reduced siRNA abundance at the hypo-DSRs, as no hyper-DSRs were found in the *nrpd1-3* mutant with even more widespread reduction of siRNAs (Fig. 2A). Nearly no hyper-DSRs were found in the *zmp-2 ZMP-GFP* versus WT comparison, confirming that the *zmp* hyper-DSRs were caused by the *zmp-2* mutation. Thus, *ZMP* inhibits siRNA biogenesis at certain genomic regions. Unlike *zmp* hypo-DSRs, the *zmp* hyper-DSRs were predominantly distributed in euchromatic regions (Fig. 2D). Among the 3205 *zmp* hyper-DSRs, only 968 overlapped with Pol IV–dependent siRNA regions (Fig. 2B and dataset S2). This suggested that *ZMP* represses siRNA production by Pol IV at these 968 regions, while, at other regions, siRNAs were not normally present or were produced by another polymerase. At two of the 968 loci, Northern blot analysis validated the increase in siRNA abundance in the *zmp-1* and *zmp-2* mutants and the absence of siRNAs in the *nrpd1-3* mutant (Fig. 3C). Moreover, no siRNAs were present in the *zmp-2 nrpd1-3* double mutant, while siRNA levels were similar between *zmp-2* and *zmp-2 shh1-1* (Fig. 3C). Thus, *ZMP* represses 24-nt siRNA biogenesis by Pol IV at these loci, while *SHH1* had no effect. At the other 2237 *zmp* hyper-DSRs, Pol IV did not produce siRNAs in the WT background (Fig. 2, B and C). To determine whether siRNAs produced in the *zmp-2* mutant at all *zmp* hyper-DSRs were Pol IV dependent, we profiled sRNAs of *zmp-2 nrpd1*-*3* and *zmp-2 shh1-1* double mutants. At all *zmp* hyper-DSRs, the levels of 24-nt siRNAs were nearly completely gone in the *zmp-2 nrpd1-3* double mutant, suggesting that Pol IV was responsible for the production of siRNAs at all *zmp* hyper-DSRs (Fig. 3D, bottom left). A mild reduction in siRNA levels was found in the *zmp-2 shh1-1* double mutant (Fig. 3D, bottom left). This, together with results in Fig. 2G, suggests that siRNA biogenesis at some of the *zmp* hyper-DSRs requires *SHH1*. Methylome analysis showed that CG, CHG, and CHH methylation was present at the *zmp* hyper-DSRs in WT, and methylation in all contexts was slightly but significantly enhanced in the *zmp-2* mutant (Fig. 3E). These results demonstrate that *ZMP* represses RdDM at certain genomic locations.

*ZMP* probably repressed Pol IV–dependent siRNA biogenesis by preventing Pol IV occupancy at these sites. In contrast to *zmp* hypo-DSRs, which showed reduced NRPD1-HA occupancy in the *zmp-2* mutant background, *zmp* hyper-DSRs showed increased NRPD1-HA ChIP signals in the *zmp* mutant background (Fig. 4E), consistent with the notion that *ZMP* reduces Pol IV occupancy at these loci to repress siRNA biogenesis. To complement this observation, we sought to determine siRNA levels at *ZMP*-repressed P4BSs (*ZMP*-rep P4BSs). We first identified 431 differentially enriched NRPD1-HA ChIP peaks in the *zmp-2* mutant as compared to WT (Fig. 4G and dataset S3), which we termed *ZMP*-rep P4BSs. These sites showed euchromatic distribution and higher siRNA levels in *zmp-2* than WT (Fig. 4, H, I, and K, and fig. S8), supporting the conclusion that *ZMP* prevents Pol IV from producing siRNAs at certain euchromatic regions. At *zmp* hyper-DSRs, ZMP-HA ChIP signals were detectable but low and did not pass the filters to be called peaks at some loci (Fig. 4E and fig. S11C). Consistent with the small effect of *SHH1* on siRNA biogenesis at *zmp* hyper-DSRs, *ZMP*-rep P4BSs had minimal overlap with *SHH1*-dep P4BSs (fig. S6D).

**Fig. 6.**
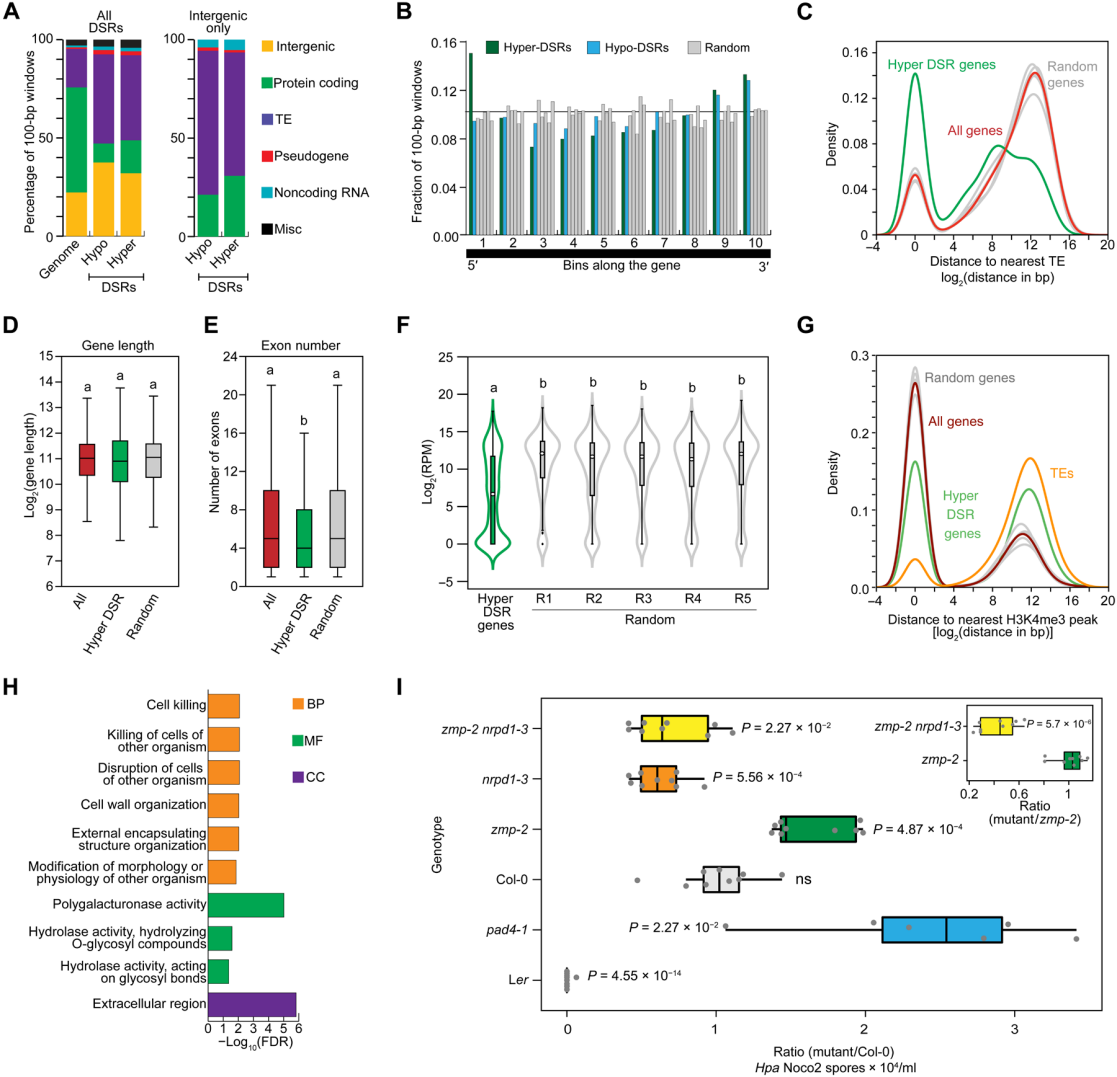
ZMP prevents Pol IV–dependent siRNA biogenesis from certain genes. (**A**) Proportions of annotated genomic features associated with the *zmp* hyper- and hypo-DSRs (left). DSR loci within intergenic regions (orange boxes) were further annotated to the closest genome features (right). Intergenic, region without any overlapping features; noncoding RNA includes miRNAs, ta-siRNAs, tRNAs, etc.; Misc, miscellaneous regions containing overlap of multiple features. (**B**) *zmp* hyper-DSRs are enriched at the 5′ end of genes. Genes overlapping the DSRs or random loci were binned into 10 segments, and counts of DSRs within each segment were tallied and represented as a fraction of the 100-bp loci. The horizontal line represents the 10% mark. (**C**) Distance of *zmp* hyper-DSR genes to the nearest TE. All genes represent all Araport11-annotated features excluding TEs (*n* = 33,452). Hyper-DSR genes, *n* = 483; randomly sampled genes, *n* = 483. (**D** to **F**) Gene length (D), exon number (E), and expression levels (F) of hyper-DSR genes compared to all genes and randomly selected genes. Statistical significant was determined by the Student’s *t* test (D and F) and Wilcoxon rank sum test (E) and represented by letters. Genotypes with the same letters show no significant differences in pairwise comparisons. (**G**) Distance of *zmp* hyper-DSR genes to the nearest H3K4me3 peaks. All genes, random selected genes, and TEs were also plotted for comparison. The “random” in (C) to (G) represents five sets of randomly selected genes with the same number as hyper-DSR genes. (**H**) Enriched gene ontology terms (FDR, *P* ≤ 0.05) of the *zmp* hyper-DSR genes. BP, biological process; MF, molecular function; CC, cellular compartment. (**I**) HpaNoco2 infection assays showing enhanced susceptibility of *zmp-2* compared to Col-0 and dependence of this effect on *NRPD1*. The comparison was also carried out between *zmp-2* and *zmp-2 nrpd1-3* (inset), and statistical evaluation methods are described in Materials and Methods.

We were interested in the nature of the loci where *ZMP* inhibits Pol IV occupancy to prevent siRNA biogenesis. As compared to *ZMP*-dep P4BSs, *ZMP*-rep P4BSs had larger numbers and fractions of protein-coding genes and intergenic regions (fig. S6F). Both *zmp* hypo-DSRs and hyper-DSRs were enriched in TEs and intergenic regions as compared to the genome, but the hyper-DSRs had a larger fraction of protein-coding genes than the hypo-DSRs (Fig. 6A). For the intergenic regions, more were close to protein-coding genes in the hyper-DSRs than hypo-DSRs (Fig. 6A). At some of these genes, 24-nt siRNAs were not produced in WT but were ectopically generated in the *zmp* mutant background (fig. S10). This led us to focus on investigating *ZMP*’s role in repressing siRNA biogenesis from genes. When all annotated genes were divided into 10 bins along their gene bodies, *zmp* hyper-DSRs overlapped with the 5′ most bin as compared to *zmp* hypo-DSRs or five sets of random sequences (Fig. 6B). Thus, *ZMP* prevents siRNA biogenesis from the 5′ most regions of certain genes. We next explored features of the genes that overlapped with *zmp* hyper-DSRs (which we termed hyper-DSR genes; dataset S4). As compared to all genes or randomly selected genes, hyper-DSR genes were located closer to TEs (Fig. 6C). While they were similar to all genes or randomly selected genes in gene length, they had fewer numbers of exons (Fig. 6, D and E). Notably, hyper-DSR genes were expressed at a lower level than randomly selected genes (Fig. 6F). H3K4me3 serves as a hallmark of the transcription start sites of actively expressed and poised genes (*35*). Genes and TEs exhibited markedly different patterns of H3K4me3, with most genes coinciding with H3K4me3 peaks while most TEs being far from the nearest H3K4me3 peaks (Fig. 6G). Consistent with the lower expression of hyper-DSR genes, these genes displayed a pattern of H3K4me3 in between genes and TEs, with a lower proportion of hyper-DSR genes coinciding with H3K4me3 peaks as compared to randomly selected genes (Fig. 6G). Thus, *ZMP* prevents Pol IV from accessing genes that have fewer numbers of exons, lie closer to TEs, and are expressed at a lower level than other genes.

### Exclusion of Pol IV from genes by *ZMP* is crucial for pathogen response

We sought to understand the biological function of *ZMP*’s role in excluding Pol IV from certain genes. The Gene Ontology terms of the hyper-DSR genes implicated their roles in plant defense against pathogens (Fig. 6H and dataset S4). Nucleotide-binding leucine-rich repeat genes (“Resistance” or R genes), defensin genes and those encoding various cell wall degradation proteins were among the hyper-DSR genes (dataset S4; the genome browser view of one example is shown in fig. S10), suggesting that the hyper-DSR genes are involved in plant defense. We examined responses of WT Arabidopsis plants (accession Col-0) and *zmp-2* to the virulent oomycete *Hyaloperonospora arabidopsidis* (*Hpa*) isolate Noco2 (Noco2). Note that Col-0 lacks the R gene *RPP5*, which mediates strong immunity against *Hpa*Noco2 (*36*, *37*). As basal defense mechanisms are intact in Col-0, this accession exhibits an intermediate level of susceptibility against virulent *Hpa* isolates, such as Noco2. The accession Landsberg *erecta* (L*er*), which is completely resistant to *Hpa*Noco2 due to the R gene *RPP5* (*37*, *38*), was used as a control. The *pad4-1* mutant, which is deficient in the basal defense regulator *PHYTOALEXIN DEFICIENT 4* (*PAD4*), was also included as a control. As expected, L*er* and *pad4-1* were more resistant and susceptible, respectively, than Col-0, as measured by the relative numbers of *Hpa*Noco2 spores produced 7 days after infection (Fig. 6I and fig. S11). The *nrpd1-3* mutant was slightly more resistant, with fewer spores than Col-0. The *zmp-2* mutant showed significantly higher numbers of spores compared to Col-0, indicating enhanced susceptibility to *Hpa*Noco2. If the enhanced *Hpa*Noco2 susceptibility of *zmp-2* were caused by increased siRNA levels at the *zmp* hyper-DSRs, then we would expect *nrpd1-3* to rescue this phenotype as the hyper-DSR siRNAs were *NRPD1* dependent (Fig. 3D, left). The *zmp-2 nrpd1-3* double mutant showed greatly reduced susceptibility to *Hpa*Noco 2 as compared to *zmp-2* (Fig. 6I, inset, and fig. S11).

## DISCUSSION

The selection of genomic targets by Pol IV is crucial in determining the genomic landscape of methylation. DNA methylation in the CHG and CHH contexts is almost exclusively associated with repeats and TEs instead of genes. In this study, we show that a PHD-containing protein, ZMP, serves as a specificity factor that recruits Pol IV to, or maintains Pol IV at, a subset of genomic sites and excludes Pol IV from some genes. ZMP was identified as a Pol IV–interacting protein on chromatin. sRNA profiling revealed that *ZMP* is required for Pol IV–dependent siRNA biogenesis at a fraction of Pol IV’s genomic targets, and the *ZMP*-dependent sites are enriched in pericentromeric regions, where Pol IV–dependent siRNA biogenesis requires *CLSY3* and *CLSY4*, but not *CLSY1*, *CLSY2*, or *SHH1*. Consistently, *ZMP* is required for Pol IV’s chromatin occupancy at these sites (Fig. 4D, *zmp* hypo-DSRs). *ZMP* also excludes Pol IV from a subset of genomic sites located on euchromatin [Figs. 2D (*zmp* hyper-DSRs) and 4H (*ZMP*-rep P4BSs)]. *ZMP* appears to protect a subset of genes from RdDM by preventing Pol IV’s occupancy at these genes. Genes protected by *ZMP* tend to be located near TEs, lowly expressed, and with fewer exons, which might be features that render them potential targets of Pol IV. Although these genes are normally lowly expressed, they may be activated by environmental stimuli (such as pathogen infection), and we predict that *ZMP*’s role in preventing Pol IV from targeting these genes might enable plants’ response to stimuli. To our knowledge, no other factors that prevent RdDM from acting on genes are known.

How does *ZMP* aid Pol IV–mediated siRNA biogenesis at some loci and inhibit it at others? We speculate that interactions between ZMP, Pol IV, and the local chromatin environment determine the effects of *ZMP* toward Pol IV. The PHD of ZMP belongs to a class that recognizes H3K4me0, and in vitro assays confirmed its preference for H3K4me0. In vitro, the PHD of ZMP also binds histone H3 tails with H3K4me1, H3K4me2, or H3K4me3 fairly well and tolerates H3K9me2. In vivo, ZMP ChIP peaks are depleted of H3K4me but are abutted by H3K4me. The in vitro and in vivo results together suggest that ZMP can bind to regions with H3K4me but has higher affinity for H3K4me-depleted regions. We noticed that both Pol IV and ZMP, and particularly Pol IV, occupy broader regions than the actual *zmp* hypo-DSRs or hyper-DSRs (Fig. 4, D and E), even when nearby DSRs were merged (fig. S9). We speculate that Pol IV can access the regions flanking these DSRs either with or without *ZMP* and transcribes into these *ZMP*-regulated regions. Given that ZMP has an SWI domain that may remodel nucleosomes and a Plus-3 domain that may associate with single-stranded DNA in the transcription bubble, ZMP perhaps aids Pol IV in transcription elongation. As Pol IV moves into the region with H3K4me0, found at ZMP-binding sites and *ZMP*-dep P4BSs (Fig. 5E), ZMP’s higher affinity for H3K4me0 together with its interaction with Pol IV stabilizes Pol IV’s chromatin association, allowing Pol IV to produce precursors to siRNAs. However, as Pol IV transcribes into the *ZMP*-rep P4BSs, the lower affinity of ZMP for the chromatin features, such as higher levels of H3K4me3 (Fig. 5E), leads to the release of ZMP-Pol IV from chromatin. Consistent with this hypothesis, ZMP ChIP signals are weak at *ZMP*-rep P4BSs and *zmp* hyper-DSRs (Fig. 4, E and G). If this model is true, then ZMP monitors local changes in H3K4 methylation status to specify Pol IV targets. The recognition of regions with H3K4me0 flanked by H3K4me3 allows ZMP to target TEs with potential transcriptional activity (i.e., high H3K4me3 at transcription start sites) in pericentromeric regions as opposed to TEs with no transcriptional activity. Conversely, at euchromatic genes that are lowly expressed and reside next to TEs, the lack of changes in H3K4me3 status in the local chromatin promotes the release of ZMP–Pol IV from chromatin. In the absence of *ZMP*, other factors, such as *SHH1*, allow Pol IV to access *zmp* hyper-DSR genes from nearby TEs.

RdDM is increasingly recognized to play a role in a variety of biological processes in addition to its role in genome stability. Despite loss of siRNA production from thousands of genomic sites, RdDM-defective *Arabidopsis* mutants have few obvious phenotypes. However, in plants with higher TE contents such as maize, tomato, *Brassica rapa*, and rice, mutants in Pol IV show developmental abnormalities (*39*–*43*), reflecting a role of RdDM in the regulation of gene expression, probably through DNA methylation at TEs near gene regulatory regions. Even in *Arabidopsis* with low TE content, Pol IV–dependent siRNAs function in sexual reproduction and responses to environmental stresses (*44*–*49*). While RdDM is increasingly recognized to regulate gene expression, our studies on *ZMP* suggest that mechanisms also exist to keep Pol IV’s activities near genes in check. Genes that *ZMP* protects from Pol IV–mediated RdDM are enriched in those involved in pathogen defense. Consistently, *zmp-2* plants are more susceptible to *Hpa*Noco2. Furthermore, enhanced susceptibility of *zmp-2* proved to be *NRPD1* dependent, suggesting that *ZMP* ensures effective defense against *Hpa*Noco2 by suppressing Pol IV activity.

## MATERIALS AND METHODS

### Plant materials and constructs

#### T-DNA mutants

All plant materials used in this study were in the Col-0 ecotype except for L*er*, which was included in the pathogen assay. Unless otherwise specified, plants were grown in a growth room under long-day conditions (16-hour light/8-hour dark) at 23°C. Newly characterized T-DNA insertion mutant lines include *zmp-1* (SALK_066029) and *zmp-2* (SALK_008955). Previously published mutant lines include *nrpd1-3* (SALK_128428), *shh1-1* (SALK_074540C), and *pad4-1* (*50*, *51*).

#### Plant expression constructs and transgenic lines

Full-length genomic sequences of *NRPD1* and *ZMP* including promoters were amplified by polymerase chain reaction (PCR) from *Arabidopsis thaliana* Col-0 genomic DNA (gDNA) using Phusion polymerase (Thermo Fisher Scientific, F530) and cloned into the entry vector pENTR/D-TOPO (Thermo Fisher Scientific, K240020). The primers are shown in table S2. The “CACC” nucleotides were added to the forward primers to aid the directional cloning into the entry vector. Reverse primers did not include the stop codon to allow epitope-tag fusion. Genes were recombined into pEarleyGate 301 (*52*) to add C-terminal HA epitopes. The same entry construct of *ZMP* was also recombined with pGWB640 (*53*), fusing *ZMP* sequences C-terminally to *YFP* to generate *pZMP::ZMP-YFP*. Constructs were transformed into the corresponding homozygous mutants via the floral dip method (*54*). Lines with a single transgene insertion were identified and bulked up for further studies. The *pNRPD1::NRPD1-3xFLAG* transgenic lines were previously characterized (*17*).

### Phylogenetic analysis of ZMP and its homologs

Protein sequences from representative plant species were downloaded from Phytozome v12 (https://phytozome.jgi.doe.gov). Homologs of ZMP were obtained using HMMsearch (*55*) and aligned by MUSCLE (*55*, *56*). A primary neighbor-joining tree was constructed by MEGA X (*57*) with default parameters to filter out false positives. Domain information of the remaining proteins was obtained from CATH/Gene3D database (www.cathdb.info). Amino acid sequences were aligned again and improved manually, and then a maximum likelihood tree with the SH-aLRT test was calculated by IQ-TREE (*58*) based on the alignment. FigTree v1.4 (http://tree.bio.ed.ac.uk/software/figtree/) was used to visualize this phylogenetic tree.

### sRNA isolation and Northern blotting

Total RNA was extracted from inflorescences by TRI reagent trademark (MRC, TR118) according to the manufacturer’s instructions. For miRNA and ta-siRNA detection, 10 μg of total RNA from each sample was resolved on a 15% urea–polyacrylamide gel electrophoresis (PAGE) gel and transferred to a Hybond NX membrane. For 24-nt siRNA detection, 200 μg of total RNA was subjected to 50% polyethylene glycol precipitation to enrich for sRNAs, which were resolved by gel electrophoresis on a 15% urea-PAGE gel and transferred to a Hybond NX membrane. The RNA was cross-linked to the membrane with *N*-(3-dimethylaminopropyl)-*N*′-ethylcarbodiimide hydrochloride (EDC) (Sigma-Aldrich, E6383) cross-linking buffer [0.16 M EDC and 0.13 M 1-methylimidazole (pH 8.0)] at 65°C for 90 min. Five prime ^32^P-labeled antisense DNA oligonucleotides were used as probes to detect miR166, tasiR255, and U6. Oligonucleotide probes used are listed in table S2. For the detection of *zmp* hypo- and hyper-DSR siRNAs, 300– to 500–base pair (bp) templates corresponding to the siRNA-generating regions were amplified by PCR from gDNA using site-specific primers (table S2). The double-stranded DNA probes were randomly labeled by ^32^P–2′-deoxycytidine 5′-triphosphate with a DecaLabel DNA labeling kit (Thermo Fisher Scientific, K0622). Probes were added to the hybridization buffer [5× SSC, 20 mM Na_2_HPO_4_ (pH 7.2), 7% SDS, 2× Denhardt’s solution:2% Ficoll (type 400), 2% polyvinylpyrrolidone, and 2% bovine serum albumin] and incubated with the membrane at 55°C overnight. After two wash steps (2× SSC and 0.1% SDS at 55°C for 20 min each time) to remove excess probes, signals were detected using a Typhoon phosphorimaging system.

### Co-IP, gel filtration chromatography, and Western blotting

The transgenic plant lines *pNRPD1::NRPD1-HA* and *pZMP::ZMP-YFP* described above were crossed. The resulting F1 plants were used for co-IP and gel filtration assays. *pNRPD1::NRPD1-HA* and *pZMP::ZMP-YFP* plants were also grown under the same conditions and used as controls.

#### Co-IP assay

Approximately 0.5 g of inflorescence was collected from each genotype and ground in liquid nitrogen into a fine powder, which was then resuspended in 2 ml of lysis buffer [50 mM tris (pH 7.5), 150 mM NaCl, 5 mM MgCl_2_, 10% glycerol, and 0.1% NP-40] containing protease cocktail inhibitors (MilliporeSigma, 4693132001). The lysate was cleared by centrifugation at 16,000*g* for 10 min at 4°C. The supernatants were incubated with 5 μl of anti-HA antibody (Sigma-Aldrich, H6908) and 30 μl of Dynabeads Protein A and G (protein A:G ratio is 1:1) (Invitrogen, 10002 and 10004) or with 10 μl of green fluorescent protein (GFP)–Trap (ChromoTek, gtma-20) at 4°C for 2 hours, under slow rotation. The beads were then washed five times for 5 min each with 1 ml of lysis buffer and resuspended in 50 μl of SDS-PAGE loading buffer. Input (15 μl) and bead eluate were used for Western blot analysis.

#### Gel filtration chromatography

One gram of inflorescence collected from F1 plants expressing both *pNRPD1::NRPD1-HA* and *pZMP::ZMP-YFP* was ground to a fine powder and resuspended in phosphate-buffered saline (PBS) buffer. One milliliter of total extracts was filtered through a 0.22 μm of filter and loaded onto a Superdex 200 10/300 GL column (GE Healthcare). Fractions (500 μl) were collected at 0.5 ml/min. To estimate the molecular weight of each fraction, a standard curve was generated using the calibration proteins Ferritin (440 kDa), γ-globulin (160 kDa), bovine serum albumin (67 kDa), and lysozyme (14 kDa). Each fraction was combined with nine volumes of ethanol for protein precipitation, and the precipitate was subsequently resuspended in 100 μl of PBS buffer. Each fraction (20 μl) was used for Western blot analysis.

The co-IP and gel filtration samples were resolved on 10% SDS–PAGE gels. The proteins were then detected by Western blotting using either the HA monoclonal antibody (Roche, 11867423001) at a dilution of 1:2000 or the GFP monoclonal antibody at a dilution of 1:2000. Goat anti-rat immunoglobulin G (IgG) horseradish peroxidase (Invitrogen, 31470) was used at a dilution of 1:5000 as the secondary antibody for the HA primary antibody, and goat anti-mouse IgG horseradish peroxidase (Bio-Rad, 1706516) was used at a dilution of 1:5,000 as the secondary antibody for the GFP primary antibody. All Western blots were developed using the ECL2 Western Blotting Substrate (Pierce, 80196).

### sRNA-seq library preparation, sequencing, and data processing

#### sRNA isolation

Inflorescences from three biological replicates of WT, *zmp-2*, *nrpd1-3*, *pZMP::ZMP-YFP/zmp-2*, *zmp-2 nrpd1-3*, and *zmp-2 shh1-1* were collected and frozen in liquid nitrogen and kept at −80°C until use. Total RNA was extracted as described above and resolved in 15% urea–PAGE gels, from which gel pieces corresponding to the 15- to 40-nt sRNA fraction were excised. The sRNAs were recovered by soaking the gel slices in 0.4 N NaCl, followed by ethanol precipitation. The resulting sRNAs were then used for library preparation with the NEBNext Multiplex Small RNA Library Prep Set for Illumina (New England Biolabs, E7300) following the user’s manual. The final library products were resolved on a 12% UREA-PAGE gel, from which the 150-bp band as determined by the pBR322 DNA-MspI Digest ladder (New England Biolabs, E7323AA, provided in New England Biolabs 7300) was excised. The libraries were pooled and sequenced (single-end 50-bp, SE50) on a HiSeq 2500 instrument (Illumina).

#### Data processing and mapping

Raw 50-nt single-end reads were subjected to adapter trimming using a custom Perl script. Trimmed reads (≥18 nt) were aligned to a custom index containing *Arabidopsis* ribosomal RNA (rRNA)/tRNA/small nucleolar RNA regions using Bowtie v1.1.0 (*59*) with the parameters “-v 2 -k 1”, and reads that aligned to the 45*S* rRNA regions were counted. Subsequently, all aligned reads were discarded and the remaining unaligned reads were mapped to the *Arabidopsis* genome (TAIR10) using ShortStack v3.4 (*60*) with parameters (--mismatches 0 --mmap u --bowtie_m 1000 --ranmax 50). Mapped reads were normalized by calculating the RPMR value (reads per million of 45*S* rRNA reads) (*4*). Published sRNA-seq dataset (GSE99694) from (*19*) was downloaded and processed following our pipeline.

#### Differential expression analysis

For DSR analysis, the genome was first divided into 100-bp nonoverlapping windows, and total count of sRNA reads within each window was obtained. Reads were assigned to only one window based on the 5′ end to reduce overcounting of reads spanning multiple windows. Counts within each window were normalized by calculating the RPMR value (*4*). Differential analysis was performed in R using the edgeR package (*61*) with a fold change of 2 and a false discovery rate (FDR) ≤ 0.05 as the cutoff criteria. Gene ontology analysis of hyper-DSR genes was carried out using AgriGO v2 (*62*) with default settings.

#### Visualization

sRNA tracks were generated using bedtools v2.26.0 (*63*) and custom Perl scripts and visualized in the Integrative Genomics Viewer (IGV) version 2.8.2 (*64*). Distribution of genes, TEs, and DSRs along the chromosomes was generated in R using karyoploteR v1.16.0 (*65*). Heatmaps of 24-nt sRNA abundance (Fig. 3D and fig. S8) were visualized using the deepTools2 suite v.3.4.0 (*66*). bigWig files containing sRNA abundance in *zmp* and WT were compared using the “bigwigCompare” tool to generate bigWig files for visualization in IGV with parameter “--skipZeroOverZero.” A data matrix was generated using the bigWig files and the “computeMatrix” tool with parameters “scale-regions -b 1000 -a 1000 --skipZeros --binSize 50.” Last, the “plotHeatmap” tool was used to visualize the dataset.

### mRNA-seq library construction, sequencing, and data processing

#### RNA isolation and mRNA-seq library construction

Inflorescence collection and total RNA isolation were as described above. Total RNA from each genotype was used to generate mRNA-seq libraries using the NEBNext Ultra RNA Library Prep Kit (New England Biolabs, E7530). All size selection and clean-up steps were performed using AMPure XP **(**Beckman Coulter, A63881). The resulting libraries were pooled and sequenced (paired-end 150 bp) on a HiSeq 2500 instrument (Illumina).

#### mRNA-seq data processing and analysis

Raw 150-nt paired-end reads were trimmed using Trim Galore (*67*) with parameters “--paired --fastqc --trimn” and mapped to the Arabidopsis genome (TAIR10) with the Araport11 gff annotations (*68*) using the STAR aligner v.2.5.3a (*69*) with the parameter “--quantMode GeneCounts.” mRNA tracks were generated using bedtools v2.26.0 (*63*) and custom Perl scripts and visualized in the IGV version 2.8.2 (*64*).

### MethylC sequencing library construction, sequencing and data processing

#### DNA isolation

Twelve-day-old seedlings were collected from WT, *zmp-2*, and *nrpd1-3* lines with two biological replicates per genotype. gDNA from these lines was isolated using the DNeasy Plant Mini Kit (QIAGEN, 69104).

#### MethylC sequencing library

Purified gDNA (5.0 μg) was used to generate MethylC sequencing (MethylC-seq) libraries as described by Lister *et al.* (2008) (*70*) with minor modifications. Briefly, gDNA was fragmented to approximately 200 bp by sonication with a Covaris sonicator according to manufacturer’s instructions (Covaris, S220) and then subjected to end repair and ligation of methylated adapters provided by Illumina (Illumina, FC-121-2001) per the manufacturer’s instructions for gDNA library construction. Adapter-ligated gDNA (100 to 200 ng) was subjected to sodium bisulfite treatment using a MethylCode bisulfite conversion kit (Thermo Fisher Scientific, MECOV50). Converted and adapter-ligated DNA fragments were enriched by 12 cycles of PCR with the following reaction composition (50-μl volume): 2.5 U of uracil-insensitive PfuTurboCx Hotstart DNA polymerase (Thermo Fisher Scientific, AM2694), 5 μl of 10× PfuTurbo reaction buffer, 25 μM deoxynucleoside triphosphates, and 3 μl of PCR primer cocktail (Illumina, FC-121-2001). The thermocyling was as follows: 95°C for 2 min, 98°C for 30 s, then 12 cycles of 98°C for 10 s, 65°C for 30 s, and 72°C for 4 min, and completed with one 72°C for 10-min step. The library was purified with a PCR purification kit (Invitrogen, K310001) and quantified on a Bioanalyzer Instrument (Agilent). The resulting libraries were pooled and sequenced (paired-end 150 bp; PE150) on a HiSeq 2500 instrument.

#### MethylC-seq data processing and analysis

Raw 150-nt paired-end reads were trimmed using Trim Galore (*67*) with parameters “--paired --fastqc --trim1.” Trimmed reads alignment, deduplication, and methylation calling were processed using bismark v0.17.0 (*71*). Methylation calls were generated using the “bismark_methylation_extractor” function with the following parameters “--ignore 5 --ignore_r2 6 --ignore_3prime 2 --ignore_3prime_r2 3 --no_overlap --comprehensive --cytosine_report –CX.” The “ignore” parameters were used to remove unwanted biases from the read ends. Output from the “CX_report” file was used to generate methylation perC tracks for each DNA context (i.e., CG, CHG, and CHH) using a custom Perl script and converted to bigWig format using “wigToBigWig” from the UCSC Genome Browser and Blat software (http://hgdownload.cse.ucsc.edu/admin/exe/linux.x86_64/). Tracks were loaded into the IGV version 2.8.2 (*64*) for visualization. Metagene plots were generated using SeqPlots v3.0.12 (*72*) with 50-bp bins.

### ChIP, ChIP-MS, and ChIP-seq DNA Affinity purification, DAP library preparation, sequencing, and data processing

#### Chromatin immunoprecipitation

An HA-tagged Pol IV line, *pNRPD1::NRPD1-HA*, in Col-0, and the *zmp-2* mutant background, as well as a *pZMP::ZMP-HA* line in Col-0, were used for ChIP-seq. ChIP was performed as described (*73*) with minor modifications. For each genotype, 2.0 g of inflorescences were collected, ground to a fine powder in liquid nitrogen, and cross-linked with 1% formaldehyde (Sigma-Aldrich, F8775) for 20 min at room temperature with slow rotation. The chromatin was then fragmented to 300 bp by sonication, and the lysate was incubated with anti-HA polyclonal antibody (Sigma-Aldrich, H6908) at 4°C overnight. Subsequently, Dynabeads Protein A and Protein G were added followed by incubation for an additional 2 hours at 4°C. The beads were washed five times for 5 min each at 4°C and eluted twice by incubation in elution buffer (1% SDS and 0.1 M NaHCO_3_) at 65°C under rotation for 15 min each time. The cross-linking was reversed by incubation at 65°C overnight, and the DNA was purified using a phenol:chloroform:isoamyl alcohol kit (Thermo Fisher Scientific, 17908). ChIP-seq libraries were prepared from the resulting DNA using the NEBNext Ultra II DNA Library Prep Kit (New England Biolabs, 7645) and sequenced (paired-end 150 bp; PE150) on a HiSeq 2500 instrument (Illumina). For H3K4me3 ChIP-seq, WT inflorescences were collected and subjected to the same procedure above except that anti-H3K4me3 antibody (Abcam, ab8580) was used.

#### ChIP-seq data processing and mapping

Raw 150-nt paired-end reads from ChIP-seq datasets (i.e., NRPD1-HA, ZMP-HA, and H3K4me3) were first trimmed using Trim Galore v0.4.3 (*67*) with parameters “--paired --fastqc –trimn.” Remaining high-quality trimmed reads were aligned to the Arabidopsis reference genome (TAIR10) using bowtie2 v2.2.9 (*74*).

#### Peak calling and differential peak analysis

For all ChIP-seq datasets, peak calling was carried out using MACS v2.2.6 (*75*) with parameters “-BAMPE -g 1.19e8 --keep-dup auto –bdg,” and input was used as the control. For NRPD1-HA ChIP (Col-0, *NRPD1-HA*, *zmp-2 NRPD1-HA*) and ZMP-HA ChIP (Col-0 and *ZMP-HA*) with two biological replicates per genotype, we identified high confident peaks from the replicates using the Irreproducibility Discovery Rate framework v2.04.2 (*76*) with default parameters. For NRPD1-HA ChIP, differentially enriched peaks (i.e., ZMP-dep and *ZMP*-rep P4BSs) between NRPD1-HA and *zmp-2* NRPD1-HA lines were determined using DiffBind v3.0.7 (*77*) with default parameters. Overlap of peak calls between NRPD1-HA and ZMP-HA samples was determined using bedtools v2.26.0 (*63*).

#### Peak visualization and analysis

NRPD1-HA, ZMP-HA, and H3K4me3 enrichment over P4BSs and 24-nt siRNA-enriched and siRNA-depleted regions (DSRs) were visualized using the deepTools2 suite v3.4.0 (*66*). For H3K4me3 ChIP, genes were derived from the Araport11 annotations (*68*). Sorted bam files (input and IP) from the bowtie2 output were compared using the “bamCompare” tool to generate bigWig files for visualization in IGV with parameter “--ignoreDuplicates.” A data matrix was generated using the bigWig files and the “computeMatrix” tool with parameters “scale-regions -b 1000 -a 1000 --skipZeros”. Last, the “plotHeatmap” tool was used to visualize the dataset. Public H3 datasets were downloaded and processed following the workflow described earlier. Accessions of public histone H3 datasets used include DRP003416 (*34*) [DRR072861 (H3), DRR072863 (H3K4me1), DRR072863 (H3K4me2), DRR072864 (H3K4me3), and DRR072865 (H3K9me2)].

#### Chromatin immunoprecipitation coupled with mass spectrometry

ChIP-MS was performed with inflorescences from WT (Col-0), *pNRPD1::NRPD1-3xFLAG*, and *pNRPD1::NRPD1-3xHA* following the procedure described in the published protocol (*21*). Anti-FLAG M2 magnetic beads (Sigma-Aldrich, M8823) and HA antibody (Sigma-Aldrich, H6908) coupled with Dynabeads Protein A and G (Protein A:G ratio is 1:1) (Invitrogen, 10002 and 10004) were used in this assay to pull down the NRPD1 protein complex.

### *Escherichia coli* recombinant protein expression, plasmid construction, and in vitro histone binding assay

#### Construction of protein expression plasmids

DNA fragments representing the ZMP PHD domain (PHDZMP, amino acids 1 to 150) or the PHD-deleted ZMP protein (ZMP-ΔPHD, amino acids 151 to 602) were cloned into a modified pET-21a vector with a 5′-end SUMO tag. These constructs, His-SUMO-PHDZMP and His-SUMO-ZMP-ΔPHD, were transformed into *E. coli* BL-21. Individual colonies were inoculated in kanamycin-containing LB medium at 37°C. Induction was performed with 0.2 mM isopropyl-β-d-thiogalactopyranoside when the bacterial optical density reached 0.6 (Sigma-Aldrich, I6758) and the cells were further grown at 18°C for 16 hours. Recombinant proteins were further purified with Ni–nitrilotriacetic acid resin (Thermo Fisher Scientific, 88222) following the manufacturer’s instructions.

#### In vitro binding assay

For histone peptide binding, 1 μg of biotinylated histone peptides was incubated with 15 μl of Dynabeads MyOne Streptavidin T1 (Invitrogen, 65601) in binding/washing buffer (50 mM tris-HCl 7.5, 300 mM NaCl, 0.5% NP-40, and 1 mM phenylmethylsulfonyl fluoride + protease inhibitors) at 25°C for 1 hour with shaking at 1100 rpm. After washing away the nonmobilized histone peptides, 1 μg of recombinant proteins (His-SUMO-PHDZMP or His-SUMO-ZMP-ΔPHD) in binding/washing buffer was added and incubated for 2 hours at 4°C with rotation. The beads were washed five times with binding/washing buffer, and the bound proteins were denatured and eluted by heating the beads at 95°C for 5 min. The proteins were subsequently resolved in 12.5% SDS-PAGE gels and analyzed by Western blotting with 6× His antibody (MilliporeSigma, 05-949) used at 1:5000 dilution.

### *Hpa*Noco2 infection assays

Infection of plants with *Hpa* isolate Noco2 was performed as described (*78*). Briefly, 2-week-old *Arabidopsis* seedlings were spray-inoculated with a suspension of *Hpa*Noco2 spores (~2 to 3 × 10^4^ spores/ml) using Preval sprayers (Preval, Coal City, IL, USA). Seven days after inoculation, plants were scored by counting spores per 20 seedlings using a hemocytometer. Spore counts were recorded from two to four biological replicates per genotype, with two or three technical repeats per replicate for each biological replicate. The *pad4-1* mutant and L*er* served as the susceptible and resistant controls, respectively. Statistical significance was determined by the one-sample Student’s *t* test with H_0_ (mu = 1) and Ha (mu ≠ 1). ns indicates not significant; *P* values for comparisons against the Col-0 control are indicated next to each genotype. For the inset of Fig. 6I, *P* value was calculated between the *zmp-2* and *zmp-2 nrpd1-3* genotypes. All statistical tests were adjusted for multiple comparisons using the Holm correction in R.

### Accession numbers

The high-throughput sequencing data generated in this paper have been deposited in the Gene Expression Omnibus database (GSE 171934).

## Supplementary Materials

### Other Supplementary Material for this manuscript includes the following:

Tables S1 and S2

Datasets S1 to S4

## Notes

View/request a protocol for this paper from *Bio-protocol*.
