# Supplementary material for: ZMP recruits and excludes Pol IV–mediated DNA methylation in a site-specific manner
Source: Sci Adv. 2022 Nov 25;8(47):eadc9454. doi: 10.1126/sciadv.adc9454 (PMC9699677; doi:10.1126/sciadv.adc9454)
Supplement: Supplementary file 1 — Figs. S1 to S11 [file sciadv.adc9454_sm.pdf]

Science Advances
AAAS

Supplementary Materials for

ZMP recruits and excludes Pol IV–mediated DNA methylation in a site-specific manner

Yuan Wang *et al.*

Corresponding author: Beixin Mo, bmo@szu.edu.cn; Xuemei Chen, xuemei.chen@ucr.edu

Sci. Adv. **8**, eadc9454 (2022)
DOI: 10.1126/sciadv.adc9454

The PDF file includes:

Figs. S1 to S11
Legends for tables S1 and S2
Legends for datasets S1 to S4

Other Supplementary Material for this manuscript includes the following:

Tables S1 and S2
Datasets S1 to S4

Fig. S1. A phylogenetic tree of ZMP-like proteins in plants

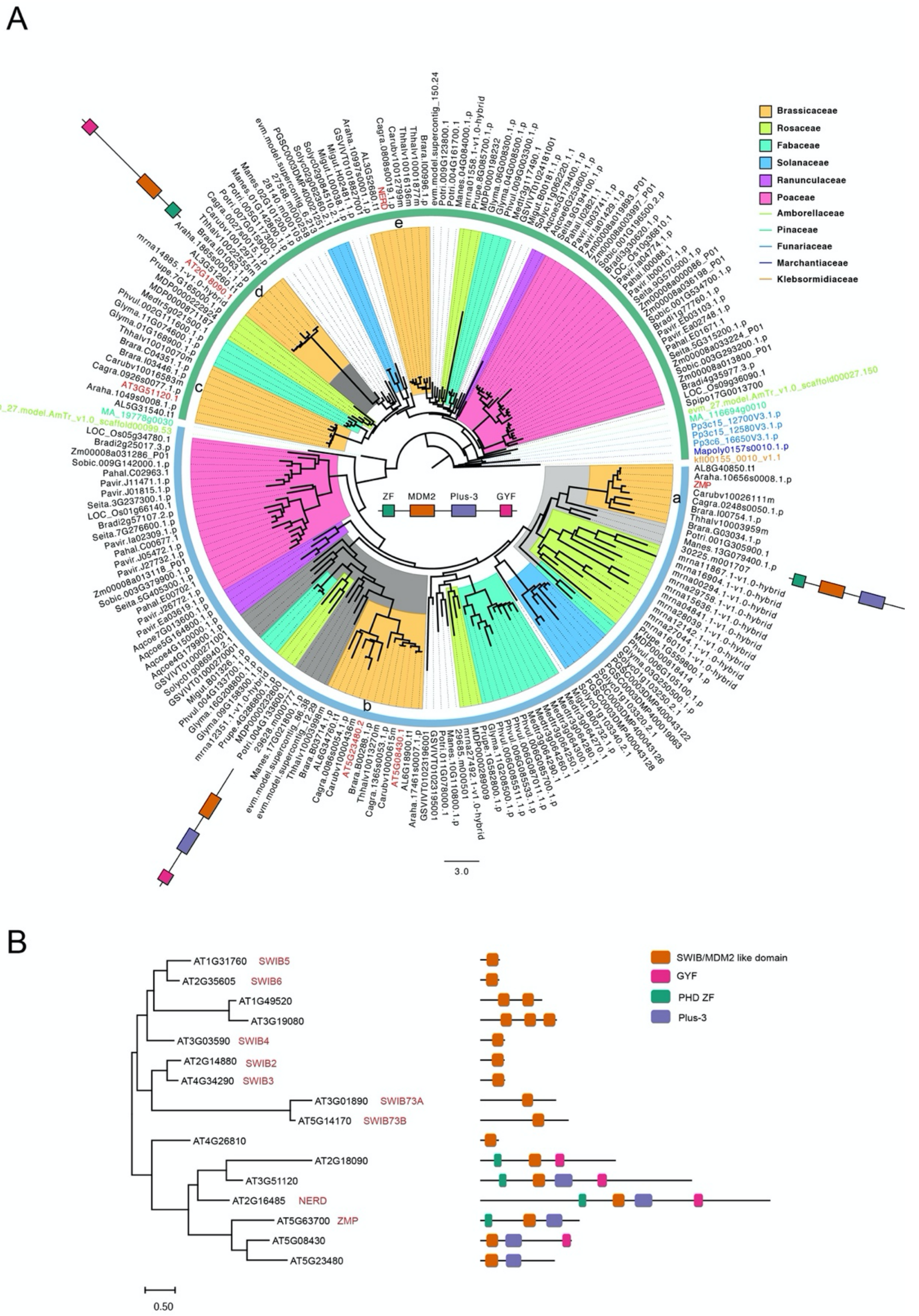

A. A maximum likelihood (ML) tree for ZMP-like proteins in representative land plants is shown in a radial format. ZMP and its Arabidopsis paralogs are highlighted in red. A simplified ancestral protein structure with representative domains of this gene family is shown in the middle. ZF, Zinc finger PHD domain; MDM2, SWIB/MDM domain; Plus-3,

Plus-3 domain; and GYF, GYF domain, which is a proline-rich domain containing a 17-aa motif (GPY[orF]xxxxM[orV]xxWxxx GYF), are shown as green, orange, purple and magenta boxes, respectively. Clades with exceptional protein structures, which are shown beside the sequence accessions, are highlighted by the grey background. Color codes for representative families of angiosperms and basal plants such as Amborella and common liverwort are shown at the top right corner. The latest common ancestor of ZMP and paralogs in multicellular plants, which contains protein domains zinc finger, MDM2/SWIB, Plus-3 and GYF, diverged into two major groups in seed plants (denoted by the green and blue bars). There would be 6 Brassicaceae clades after the γ whole-genome duplication in eudicots. But one copy was lost, resulting in five paralogous groups, indicated with lowercase letters (a through e). During evolution, the gene structure evolved, and some ancestral genes lost certain domains. For instance, the ancestor of ZMP in the latest common ancestor of rosids lost the GYF domain, and the ancestor of AT5G23480 and AT5G08430 in the latest common ancestor of core eudicots lost the zinc finger domain. More recently, the ancestor of AT2G18090 in the latest common ancestor of crucifers lost the Plus-3 domain.

B. A maximum likelihood (ML) tree and protein domain annotation for ZMP-like proteins and SWIB/MDM2 domain-containing proteins in *Arabidopsis thaliana*.

Fig. S2. Scatter plots showing the reproducibility of small RNA-seq among the three biological replicates in four genotypes as indicated

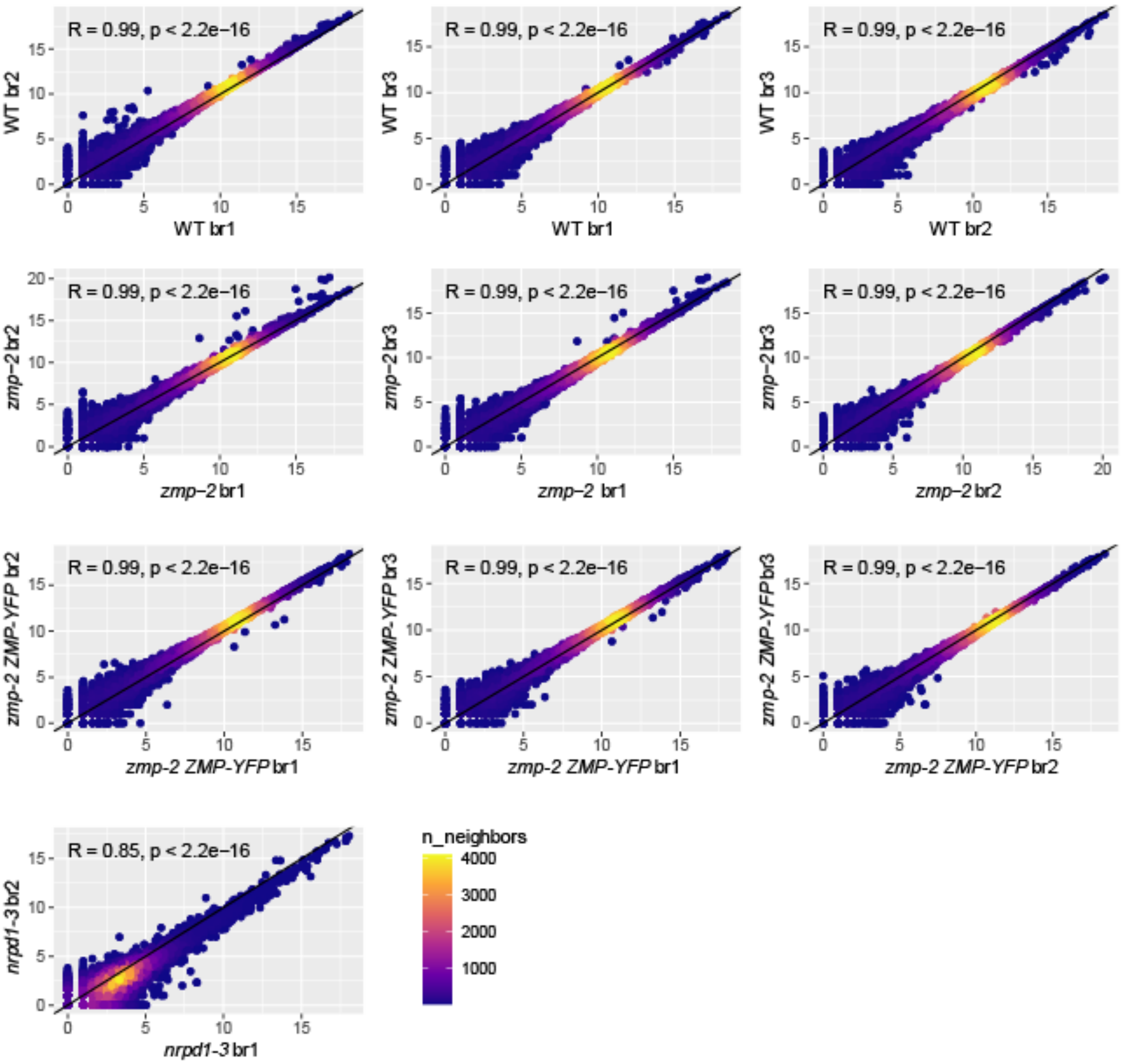

The genome was divided into 5kb bins and total small RNA counts were tallied for each bin. Scatter density plots were generated with bin density indicated by the heatmap. Pearson correlation coefficient R and p-value were calculated in R using the stat cor package. br, biological replicate.

Fig. S3. The *zmp* mutation does not affect global small RNA abundance

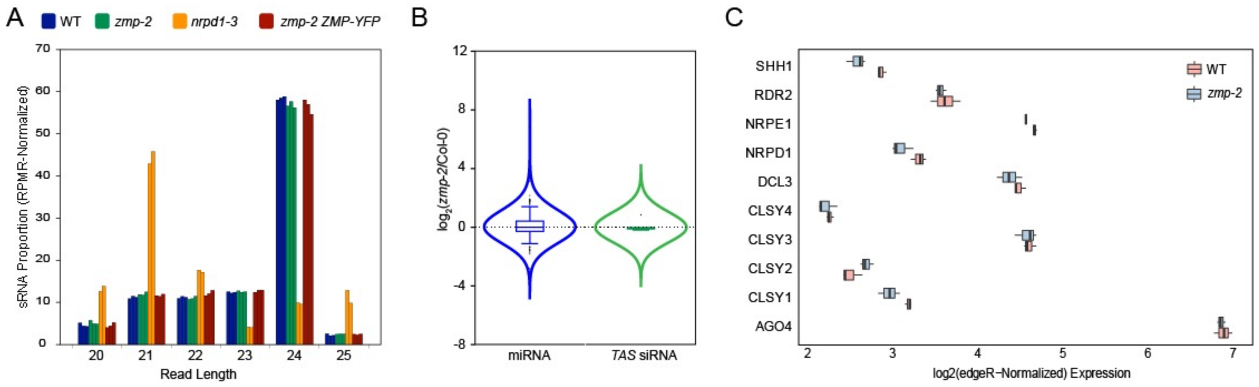

A. Relative proportions of small RNAs in each size category (from 20-25nt) in total small RNAs from four genotypes as indicated.

B. Total miRNA and ta-siRNA levels are not affected in the *zmp-2* mutant. MicroRNA and ta-siRNA abundance is represented as log2 ratios of mutant to wild type. The ta-siRNAs were those from *TAS1A*, *1B*, and *1C*, *TAS2*, *TAS3*, and *TAS4* loci.

C. Gene expression level of major RdDM components were largely unaffected in *zmp-2* mutant.

Fig. S4. Scatter plots showing the reproducibility of MethylC-seq among the biological repeats in three genotypes as indicated

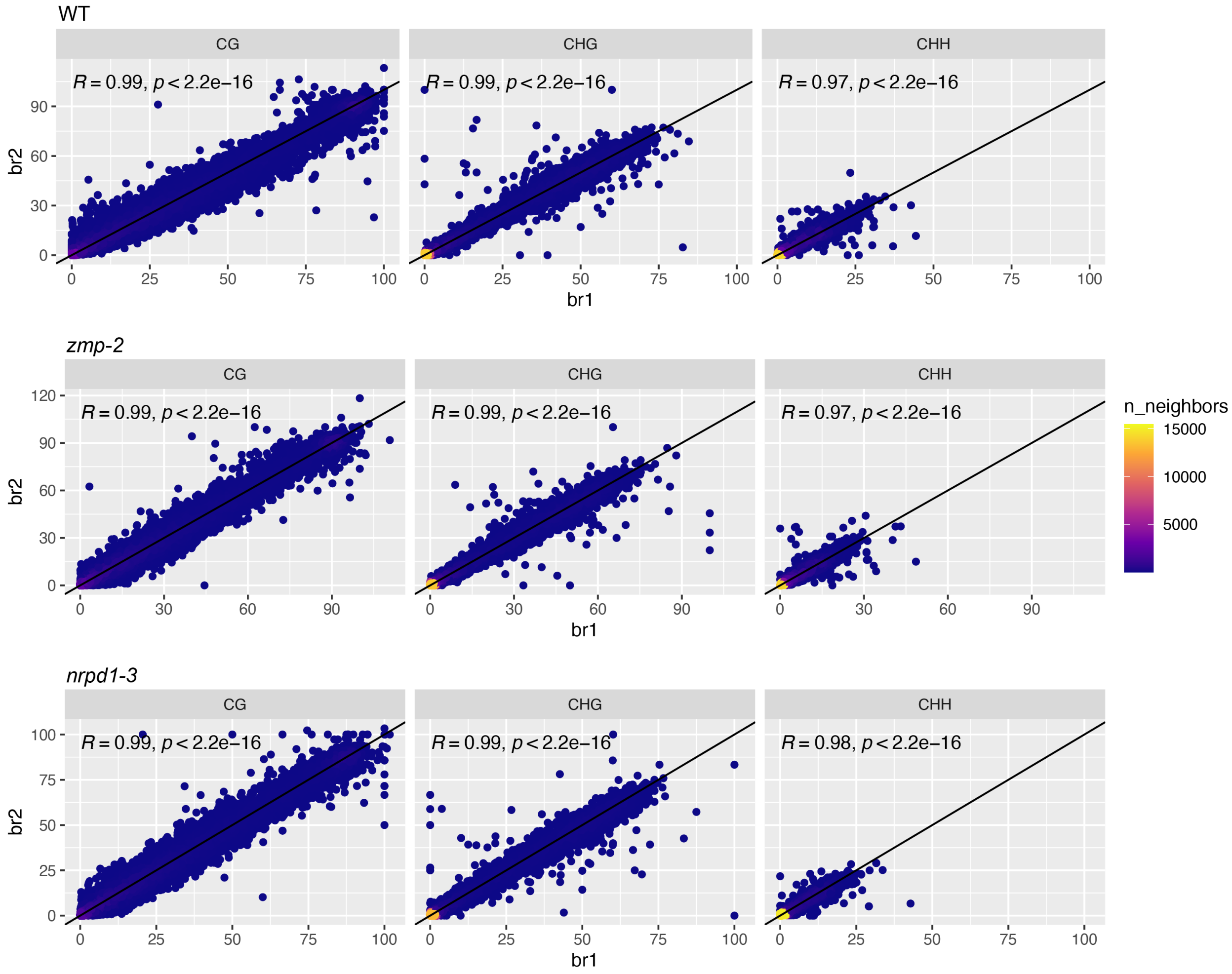

The genome was divided into 10 kb bins and the methylation level in each bin was calculated for each sequence context (CG, CHG, CHH where H = A, T, or C). Methylation level was calculated as [C/(C+T)] x 100 where C and T represent the counts of methylated and unmethylated cytosines, respectively. Scatter density plots were generated with bin density as indicated by the heatmap. Pearson correlation coefficient R and p-value were calculated in R using the stat cor package. br, biological replicate.

Fig S5. DNA methylation profiles at TE and gene regions

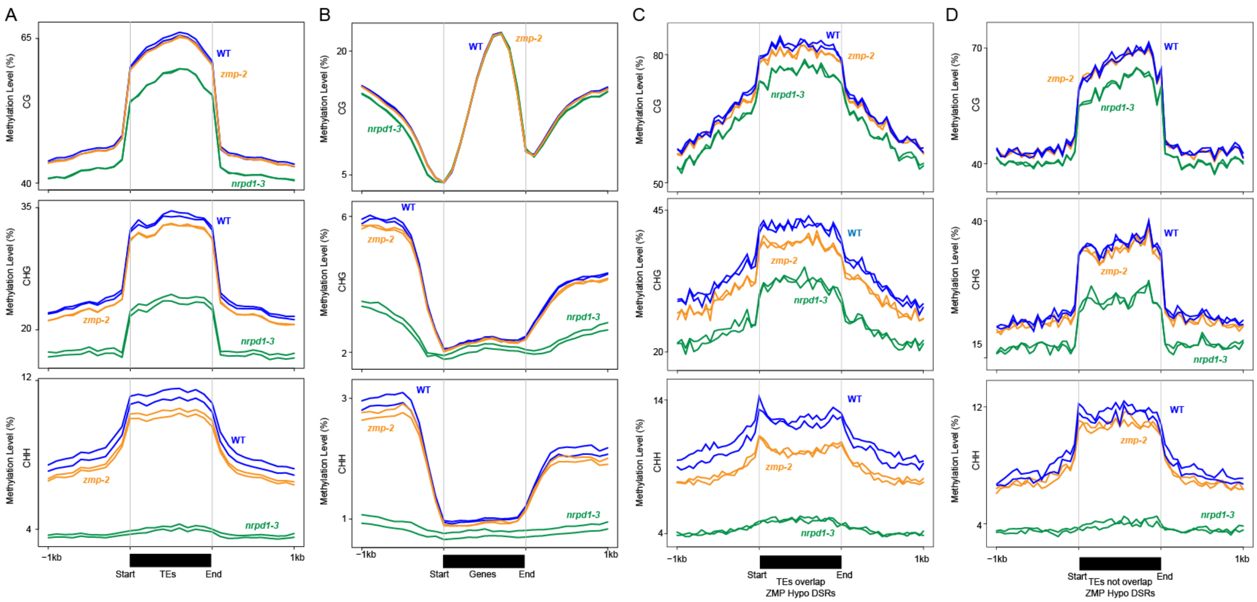

DNA methylation profiles at TE (A), gene (B) regions and a subset of TEs overlapping (C) or not overlapping (D) with ZMP hypo DSR loci in WT, zmp-2, and nrpd1-3, in the CG (top row), CHG (middle row) and CHH (bottom row) sequence context. TEs not overlapping with the ZMP hypo DSR loci were randomly selected to match the TE number in (C). Both biological replicates were plotted for each genotype (indicated by lines of the same color). H= A, T, or C. Genes and TEs were based on Araport11 annotations. Metagene plots were generated using SeqPlots v3.0.12 (*70*) with 50 bp bins.

Fig. S6. ZMP binding sites, ZMP-dependent and ZMP-repressed Pol IV binding sites are not significantly overlapped with SHH1-dependent Pol IV binding sites

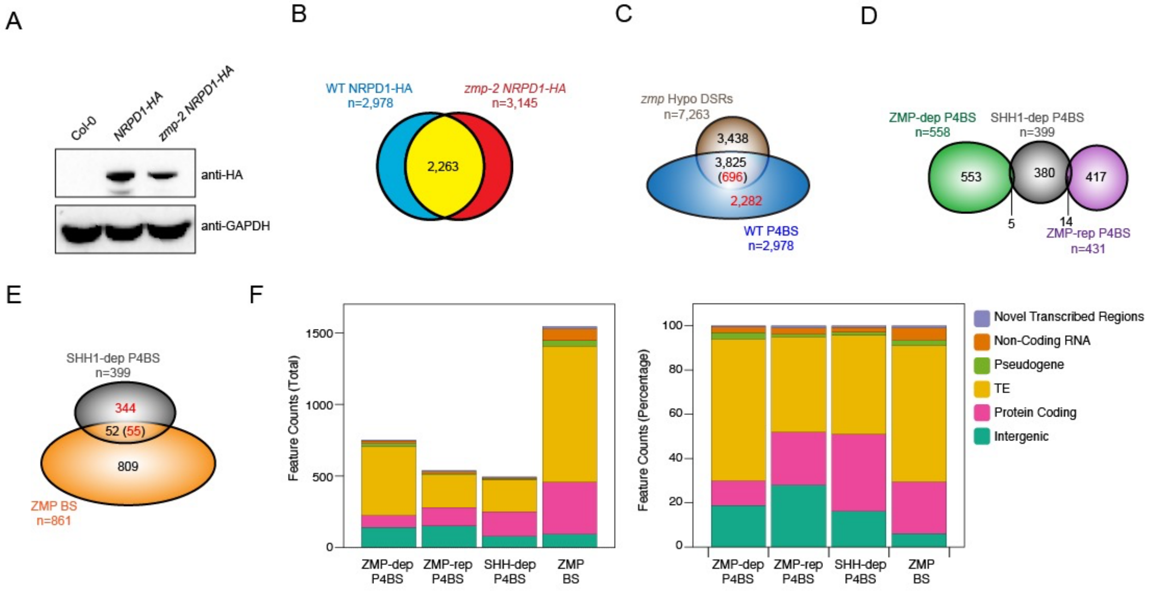

A. Immunoblot detecting the NRPD1-HA protein in WT (Col-0), *NRPD1-HA* and *NRPD1-HA zmp-2* backgrounds. GAPDH was an internal control.

B. Overlap of Pol IV binding sites (P4BS) in NRPD1-HA and *zmp-2* NRPD1-HA ChIP-seq datasets.

C. Overlap of *zmp* hypo DSRs and P4BS in WT. In the overlapping region, since the numbers of DSRs and Pol IV binding sites are different, the black and red numbers denote the number of DSRs and Pol IV binding sites, respectively.

D. Overlap analysis showing the relationship among ZMP-dependent, SHH1-dependent, and ZMP-repressed P4BS. "n" represents the number of each defined Pol IV binding regions. SHH1-dependent P4BS were obtained from Law et al. 2013(*16*).

E. Overlap of ZMP binding sites (ZMP BS) and SHH1-dependent P4BS. The numbers in red and black denote the number of SHH1-dependent P4BS and ZMP binding sites, respectively.

F. Total counts (top) and percentages (bottom) of the annotated genomic features associated with the ZMP BS and defined P4BS. These regions were determined based on Araport11 annotations. Intergenic, regions that do not overlap any defined features. Note: A binding site can overlap with

multiple features and all features were included in this plot. Therefore, the total feature counts are greater than the number of binding sites.

Fig. S7. Reproducibility of the ChIP-seq datasets

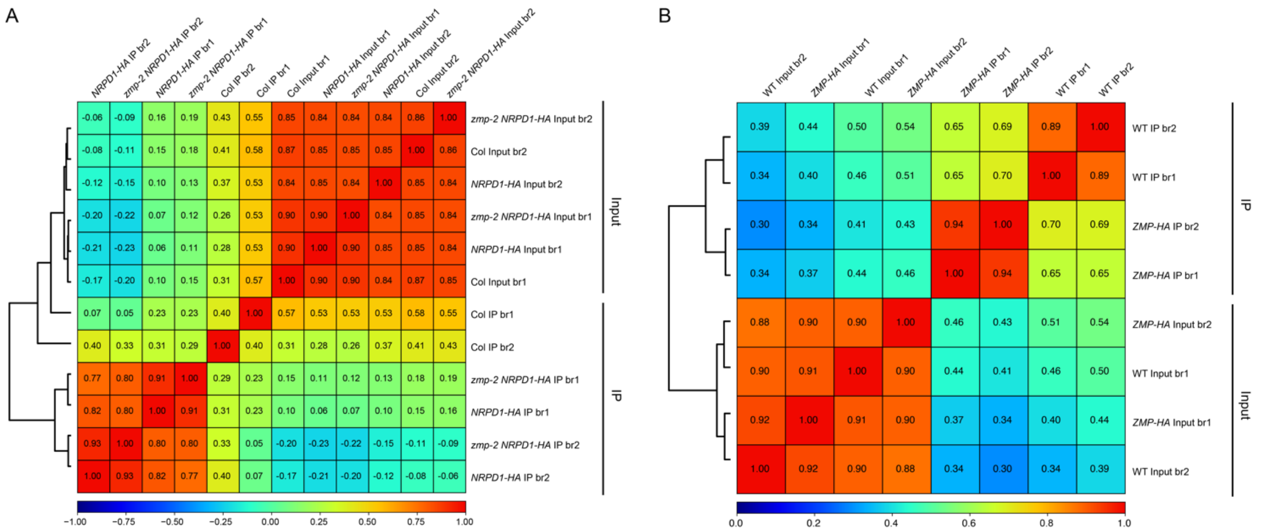

Heatmaps with Spearman correlation coefficients represented by numbers within each box for the NRDP1-HA ChIP-seq (A) and ZMP-HA ChIP-seq (B) datasets. The genome was divided into 5 kb bins and counts per bin were tallied. Bin counts and heatmaps were generated using the DeepTools analysis suite (*66*). The heatmaps show high reproducibility of the biological replicates (br) and good separation of the input and immunoprecipitated (IP) samples.

Fig. S8. Relative abundance of 24-nt siRNAs at ZMP-dep and ZMP-rep P4BS

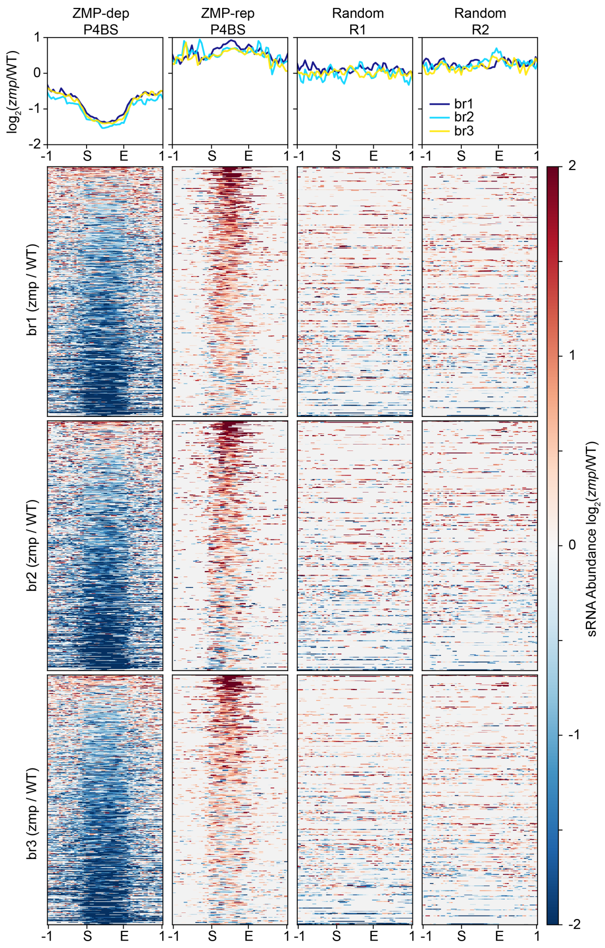

Heatmaps and profile plots for each biological replicate show signals (log2 ratios of *zmp-2*/WT in abundance of 24-nt siRNAs in 50bp bins) at the start (S) and end (E) of the indicated regions plus 1kb upstream (-1) and downstream (1). Random sites (R1 and R2) as negative controls were generated at similar size and numbers as ZMP-dep and ZMP-rep P4BS. Three replicates of WT and *zmp-2* sRNA-seq data were plotted.

Fig. S9. NRPD1-HA and ZMP-HA ChIP-seq signals at DSR loci

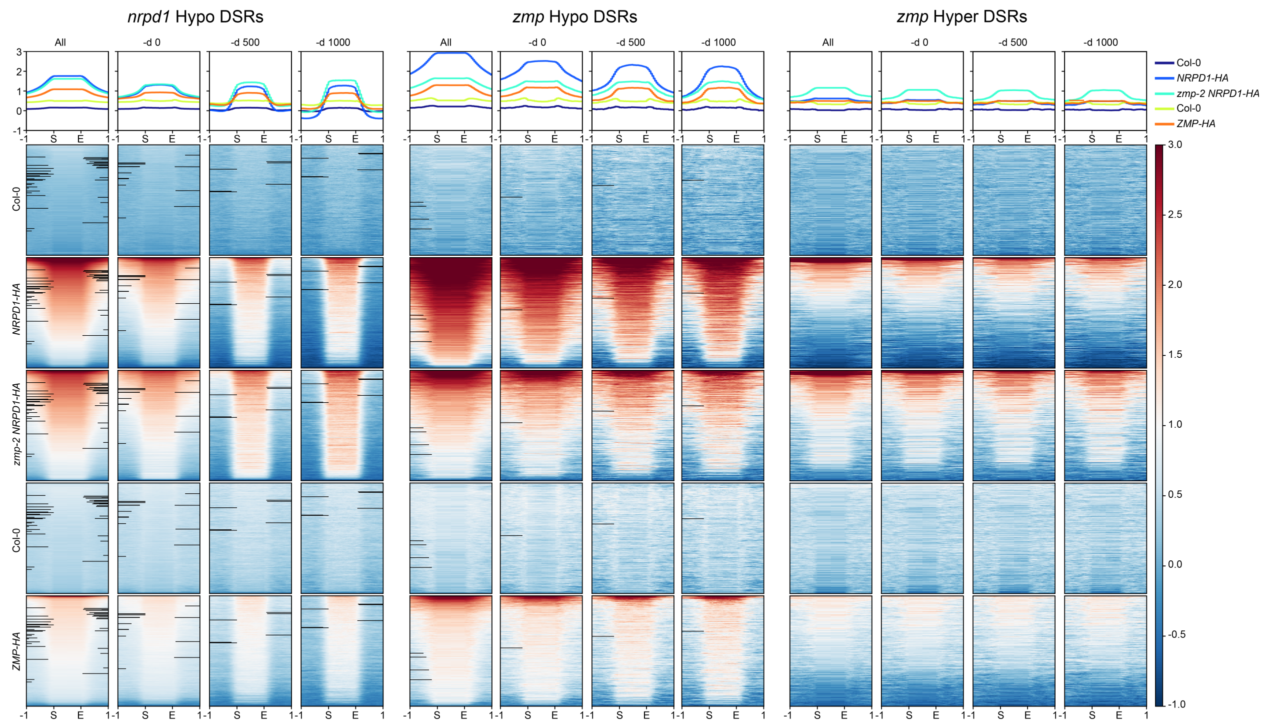

100-bp DSR regions were merged using the bedtools 'merge' function (*63*) with varying -d values indicating distance between two regions required for merging. -d 0, 500, 1000 represent merging of DSRs that are 0, 500, and 1000 bp apart, respectively. "All" represents all 100-bp windows without merging. Heatmaps and abundance profile were generated for all and merged DSR regions using the deepTools package(*66*). Plots were generated to include the start (S) and end (E) positions of each region extending 1kb upstream (-1) and downstream (1) from the start and end positions, respectively.

Fig. S10. Genome browser views showing 24-nt siRNAs are ectopically expressed in the *zmp-2* mutant at protein-coding genes.

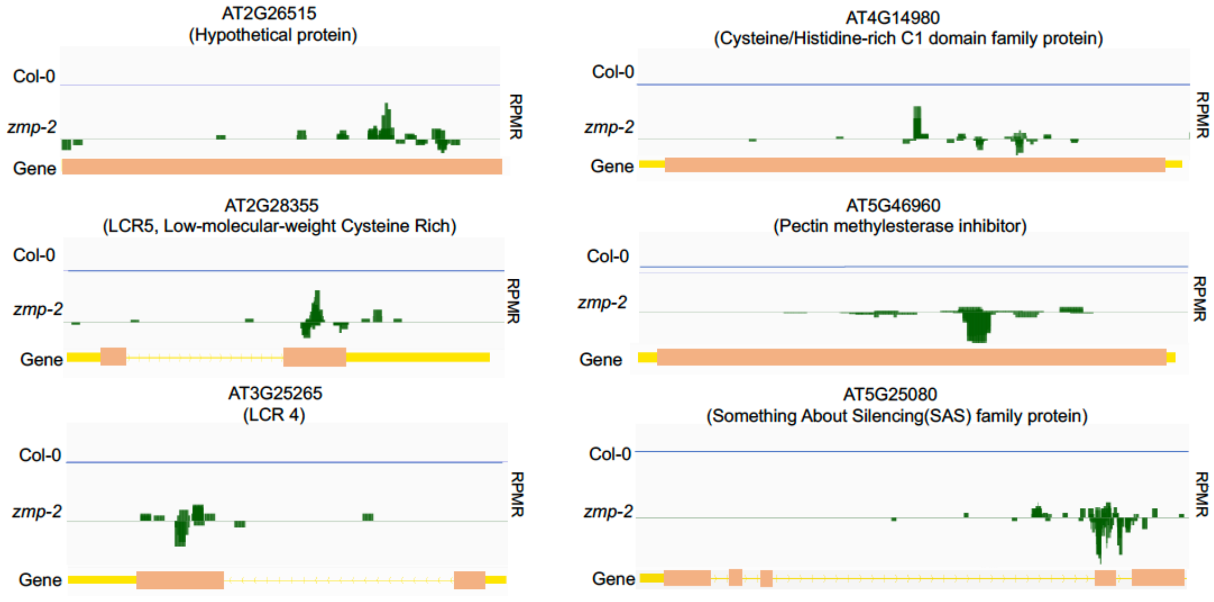

Exons of the genes are depicted by orange boxes. Scales for the siRNA tracks are from -100 to 100.

Fig. S11. *Hpa*Noco2 infection assays showing enhanced susceptibility of *zmp-2* compared to Col-0, and dependence of this effect on *NRPD1*

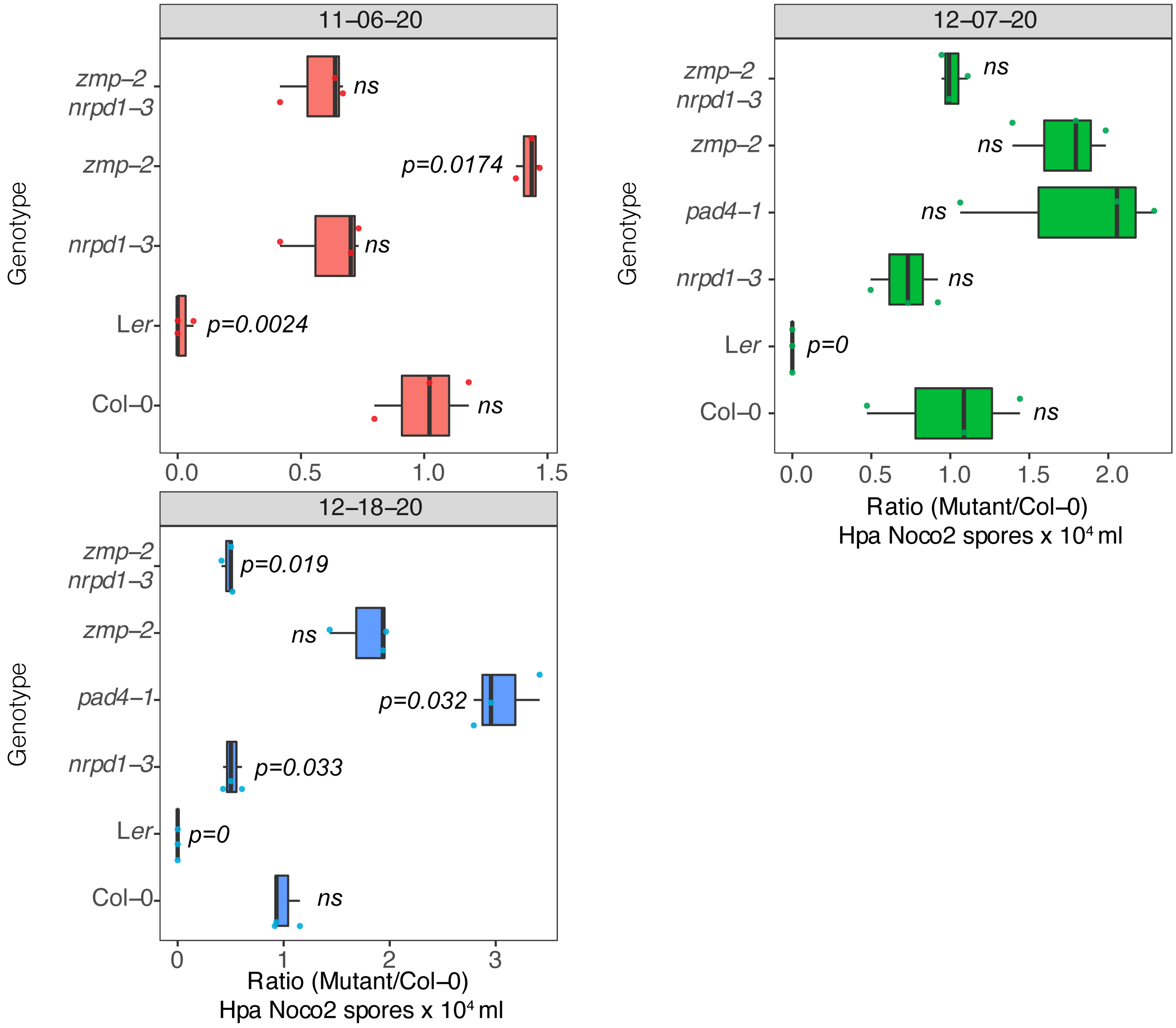

Three independent *Hpa*Noco2 infection assays (A-C) showing enhanced susceptibility of *zmp-2* compared to Col-0. The dates of experiments being performed were labelled at the corner of each plot. The *pad4-1* mutant and L*er* served as the susceptible and resistant controls, respectively. The experiment on 11-06-20 did not include the *pad4-1* genotype. The data in each plot represent three technical replicates. Statistical significance was determined by a one-way ANOVA with a post-hoc Tukey HSD test in R. Significant p values ($p < 0.05$) are indicated for pairwise comparisons against Col-0. ns, not significant.

Table S1. List of proteins identified by ChIP-MS (Separate file).

Table S2. Primers and Probes (Separate file).

Dataset S1. Resource Accessions (Separate file).

Dataset S2. DSRs (Separate file).

Dataset S3. ChIP-seq Peaks (Separate file).

Dataset S4. Hyper DSR Genes (Separate file).
